# Trafficking of the human Na^+^/H^+^ antiporter NHA2 to the plasma membrane requires cornichon COPII cargo receptors

**DOI:** 10.1002/pro.70492

**Published:** 2026-02-12

**Authors:** Karolína Kacovská, Klára Papoušková, Gal Masrati, Paul Rosas‐Santiago, Tereza Przeczková, Veronika Žárská, Nir Ben‐Tal, Olga Zimmermannová

**Affiliations:** ^1^ Laboratory of Membrane Transport Institute of Physiology of the Czech Academy of Sciences Prague Czech Republic; ^2^ School of Neurobiology, Biochemistry and Biophysics, George S. Wise Faculty of Life Sciences Tel‐Aviv University Tel‐Aviv Israel; ^3^ Instituto de Biotecnología, Universidad Nacional Autónoma de México, Av. Universidad 2001 Cuernavaca Morelos México

**Keywords:** cation homeostasis, CNIH, COPII cargo receptor, cornichon, Erv14, Na^+^/H^+^ antiporter, Nha1, NHA2, Sec24

## Abstract

A key prerequisite of transporter proteins' function is their trafficking to the target cellular membranes where they fulfill distinct physiological roles. Cornichon proteins (CNIH/Erv14) represent a highly conserved family of coat protein complex II (COPII)‐coated vesicle cargo receptors that facilitate the exit of numerous transporters from the endoplasmic reticulum (ER) to proceed via the secretory pathway. Despite their biomedical significance, the cargo specificities of the four human cornichons (CNIH1‐4) remain largely unexplored. Here, we conducted a bioinformatics analysis of the CNIH/Erv14 family, revealing evolutionary conservation profiles of the family based on an alignment of 1879 sequences. AlphaFold3 modeling predicts that residues identified as the most evolutionarily conserved in cornichon family interact with Sec24 proteins of COPII vesicles. We also demonstrate the suitability of the model yeast *Saccharomyces cerevisiae* for studying the properties and putative interactors of human cornichons. We engineered *S. cerevisiae* strains in which the endogenous cornichon gene (*ERV14*) was replaced with human CNIH1, CNIH2, or CNIH4 coding sequences or CNIH coding sequences were expressed from multi‐copy plasmids. The studied human cornichons were functional in *S. cerevisiae* cells and, to varying extents, complemented the differing phenotypes related to yeast *Sc*Erv14 roles in monovalent‐cation homeostasis. The presence of human CNIHs supported the functioning of the yeast plasma‐membrane Na^+^, K^+^/H^+^ antiporter Nha1, a known cargo of *Sc*Erv14. Both yeast *Sc*Erv14 and human CNIH cornichons improved the plasma‐membrane targeting and functioning of the human Na^+^/H^+^ antiporter NHA2 in yeast cells, identifying NHA2 as a novel cargo of cornichon COPII cargo receptors.

## 1 INTRODUCTION

The maintenance of alkali‐metal‐cation homeostasis is crucial for the proper functioning of cells of both uni‐ and multicellular organisms (Arino et al., 2010; Bernal et al., 2023; Palmer & Clegg, 2016). Typically, cells maintain a stable and neutral cytosolic pH and actively accumulate a high (approx. 100–300 mM) concentration of the main intracellular cation, K^+^ (Arino et al., 2010; Casey et al., 2010; Milo & Phillips, 2015). Potassium is essential for many physiological functions, including compensation of the negative charges of many macromolecules, the regulation of intracellular pH and cell volume, or maintenance of stable plasma membrane potential. On the contrary, high intracellular levels of Na^+^ are generally toxic for cells; however, Na^+^, being the major cation in the extracellular space in mammals (100–200 mM is present in blood plasma; Milo & Phillips, 2015), is indispensable for several cellular processes (e.g., the secondary active transport), as well as for the proper functioning of various tissues and organs. Thus, monovalent cation transporters, which strictly regulate the intracellular concentrations of Na^+^, K^+^ and H^+^ by ensuring their fluxes across both plasma membrane and membranes of intracellular organelles, are of great importance for the fitness of eukaryotic cells/organisms. In humans, the malfunctioning of alkali‐metal‐cation transporting proteins results in various diseases, including metabolic or neurological disorders and cancer (Palmer & Clegg, 2016; Pedersen & Counillon, 2019).

**FIGURE 1:**
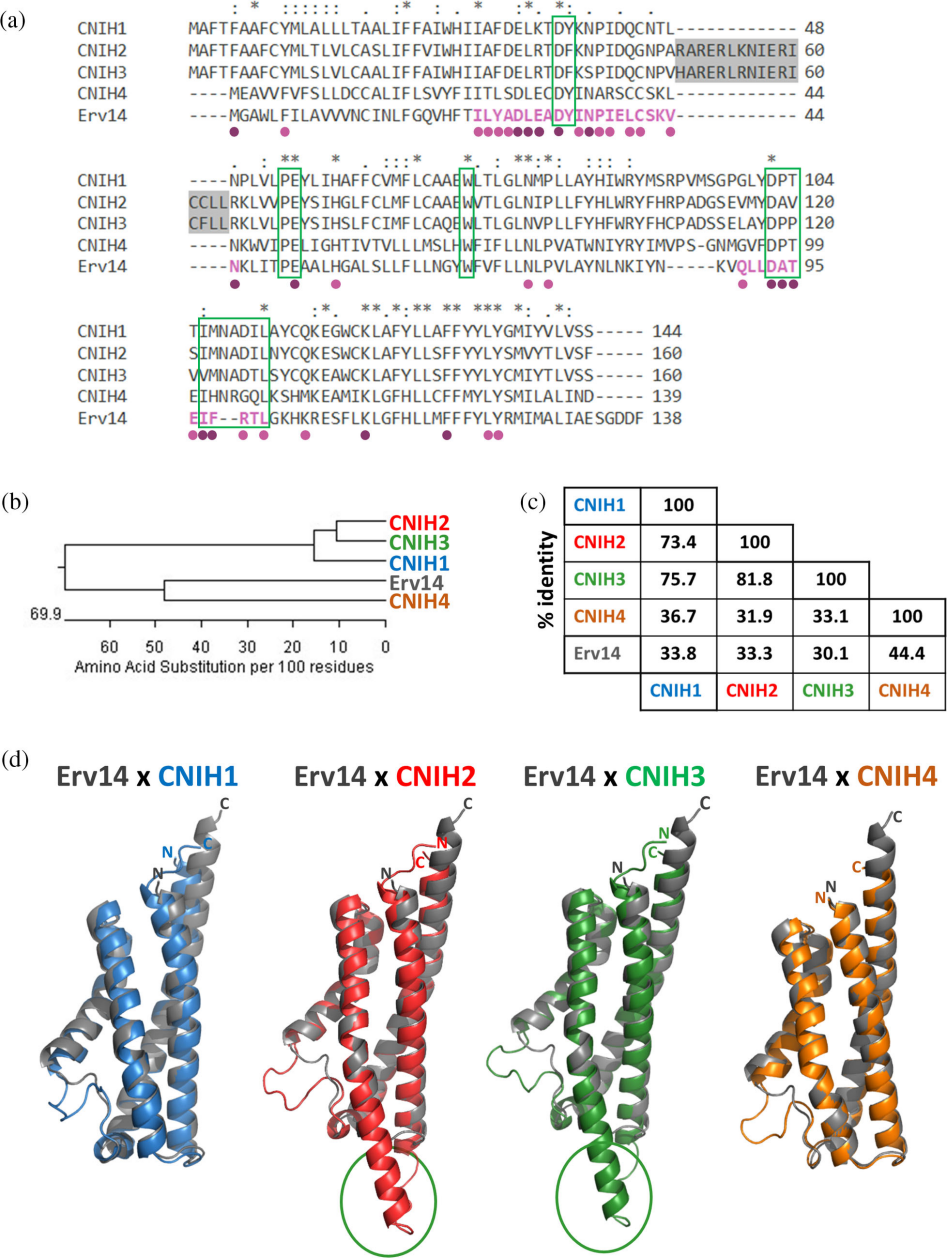
Sequence and structure analysis of *S. cerevisiae* Erv14 and human cornichons. (a) Multiple sequence alignment of yeast Erv14 and human cornichons. Gray background highlights regions typical for CNIH2 and CNIH3, green rectangles show regions residues that are discussed in detail in the text, bold magenta letters highlight Erv14's stretches of evolutionarily conserved amino‐acid residues and dots depict Erv14's amino‐acid residues with high ConSurf scores; (light dot): residue with ConSurf scale 8, (dark dot): residue with ConSurf scale 9. (*): fully conserved residue in Erv14 and human CNIHs, (:): conservation between groups of strongly similar properties, (.): conservation between groups of weakly similar properties. (b) Phylogenetic tree of *Sc*Erv14 and human cornichons. (c) Identity (%) of *Sc*Erv14 and human cornichons. (d) Overlaps of structural models of *Sc*Erv14 (grey) and human CNIHs. The prolonged beginning of the second α‐helical structure of CNIH2 and CNIH3 is highlighted with green ovals.

To fulfill their physiological roles, newly synthesized monovalent cation transporters must be properly delivered to their target membrane. Cornichon (CNIH/Erv14) proteins are a family of COPII‐coated vesicle cargo receptors that are conserved from yeasts to mammals (Figure 1, Papouskova, Cerna, et al., 2024; Papouskova, Zimmermannova, & Sychrova, 2024). Due to their ability to bind both cargoes and COPII‐complex proteins (Pagant et al., 2015; Powers & Barlowe, 2002), cornichons help their interaction partners to be incorporated into COPII‐coated vesicles and thus be efficiently exported from the ER to the Golgi apparatus and, hence, proceed via the secretory pathway. While *Saccharomyces cerevisiae* Erv14 is the most characterized of cornichon proteins (see below), the experimental discovery of cornichons' structure was possible due to the unique properties of two of the four cornichon proteins (CNIH1‐4) that are encoded by mammalian genomes. Of these, CNIH2 and CNIH3 are binding partners of AMPA‐type ionotropic glutamate receptors (AMPARs) that play a key role in excitatory synaptic transmission in the central nervous system (Royo et al., 2022; Schwenk et al., 2009). Very differently from what was found so far for other cornichon proteins, CNIH2 and CNIH3 remain in complex with AMPARs after their ER exit, and act as the receptors' auxiliary subunits in the plasma membrane (Certain et al., 2023; Coombs et al., 2012; Harmel et al., 2012; Schwenk et al., 2009; Shanks et al., 2014). This unusual permanent interaction of cornichons with AMPARs enabled cryoelectron‐microscopy studies of CNIH2/3 structures in the receptors' protein complex (Gangwar et al., 2023; Nakagawa, 2019; Yu et al., 2021; Zhang et al., 2021). The cornichons share a four‐transmembrane‐domain arrangement (Figure 1) with both N‐ and C‐termini oriented to the extracellular space (or the lumen of ER/Golgi apparatus). The majority of CNIH2/3 is buried in the membrane, with only small portions of the cornichon proteins located in the cytoplasm (Gangwar et al., 2023; Nakagawa, 2019; Yu et al., 2021; Zhang et al., 2021).

The first binding partners of the *S. cerevisiae* cornichon Erv14, which was originally found in isolated ER‐derived vesicles (Powers & Barlowe, 1998), were identified based on the phenotypes of *erv14Δ* cells. As an example, a defect in the bud selection of cells lacking Erv14 led to the discovery of Axl2, a protein required for an axial budding pattern, as a cargo of Erv14 (Powers & Barlowe, 1998). As Erv14 helps its interaction partners to exit the ER and proceed via the secretory pathway, its cargoes are typically partially accumulated in the ER of cells lacking Erv14 (Herzig et al., 2012; Powers & Barlowe, 1998). This phenomenon was utilized in the “PAIRS” (pairing analysis of cargo receptors) method, a more systematic approach to identifying cargo spectra of individual yeast cargo receptors. It revealed Erv14 to play a role in the proper targeting of a large proportion (≈32%) of the examined plasma‐membrane proteins (Herzig et al., 2012). To date, approximately 40 cargoes of Erv14 with diverse physiological functions have been discovered, including several monovalent‐cation‐transporting proteins.

Interestingly, binding partners of Erv14 do not seem to share any amino‐acid motifs which would be recognized by the cornichon protein. Instead, since the transmembrane segments of integral membrane proteins that reside in post‐Golgi compartments are longer (Sharpe et al., 2010), the length of membrane‐spanning segments of Erv14's cargoes was suggested to be responsible for the recognition of Erv14's cargoes by the cornichon. This theory was supported by experiments in which the only transmembrane segment of Mid2, a cell wall stress sensor and known Erv14 cargo (Herzig et al., 2012), was replaced with chains of leucines that differed in their lengths (14–26 amino‐acid residues, with two‐amino‐acid increments). Importantly, only the longer versions of Mid2 had an ER exit that was efficient and dependent on the presence of Erv14 (Herzig et al., 2012).

In our recent works, we have found that the deletion of the *ERV14* gene affects several physiological parameters in *S. cerevisiae* cells connected to monovalent‐cation homeostasis, including intracellular pH, cell volume, plasma‐membrane potential, tolerance to the presence of high Na^+^, K^+^ and cationic‐drug concentrations, or the ability to cope with low levels of K^+^ in media (Rosas‐Santiago et al., 2016; Zimmermannova et al., 2019). In agreement with the apparent complex role of Erv14 in the maintenance of monovalent‐cation homeostasis, three housekeeping plasma‐membrane alkali‐metal‐cation transporting proteins have been identified as cargoes of Erv14. The Na^+^, K^+^/H^+^ antiporter Nha1 (Banuelos et al., 1998; Prior et al., 1996) was the first alkali‐metal‐cation transporter whose ER exit was proved to be dependent on the interaction with Erv14 (Herzig et al., 2012; Rosas‐Santiago et al., 2016). In the absence of the cornichon protein, Nha1 is partially accumulated in the ER and, consequently, its lower amount in the plasma membrane results in a decreased ability of cells to grow in the presence of high Na^+^ levels and in a lower Na^+^ efflux from cells. In addition to Nha1, Erv14 promotes the ER exit of two K^+^‐specific transporters (Zimmermannova et al., 2019). A partial ER accumulation of Trk1, *S. cerevisiae*'s main K^+^ importer (Gaber et al., 1988; Ko & Gaber, 1991), in cells lacking Erv14 is in agreement with a lower ability of *erv14Δ* cells to grow under conditions when K^+^ is scarce in the environment. Besides Trk1, Erv14 serves as a COPII cargo receptor of the voltage‐gated K^+^ channel Tok1 (Bertl et al., 1993; Ketchum et al., 1995), whose plasma‐membrane targeting is also affected in *erv14Δ* cells (Zimmermannova et al., 2019).

The malfunctioning of CNIH proteins could have a profound effect on human health; besides the connection of cornichons to neurological disorders, such as schizophrenia (Drummond et al., 2012), CNIH proteins are also among prognostic markers and potential therapeutic targets in various types of cancer (Fang et al., 2023; Kasavi, 2022; Mishra et al., 2019; Wang et al., 2021; Wang et al., 2023; Xiao et al., 2023; Xu et al., 2025; Zhang et al., 2023). However, not much information is available on the cargo repertoires and thus physiological roles of mammalian CNIH proteins. In addition to the abovementioned ability of CNIH2/3 to promote ER exit and modulate the functioning of AMPA receptors, CNIH1 is known to play a role in the trafficking and maturation of TGFα (Castro et al., 2007), and CNIH4 was shown to be involved in the proper targeting of G‐protein coupled receptors (Sauvageau et al., 2014).

Several experiments, in which human or plant cornichons or plant cation transporters were produced in yeast cells either lacking or possessing Erv14, suggest that the way in which cornichons recognize their binding partners might be at least partially evolutionarily conserved (Castro et al., 2007; Rosas‐Santiago et al., 2015; Rosas‐Santiago et al., 2017; Wudick et al., 2018). Thus, taking into account the possibility of heterologously expressing functional mammalian alkali‐metal‐cation transporters in *S. cerevisiae* cells, together with easy yeast genetic manipulation, in this study, we employed yeast cells for studying and enriching our knowledge of human CNIH proteins and identifying their new interaction partners, especially among monovalent‐cation‐transporting proteins. To achieve this, we prepared a set of *S. cerevisiae* strains with cDNAs encoding individual human CNIH proteins (CNIH1, CNIH2, and CNIH4) either integrated into the genome and replacing the *ERV14* gene or inserted in multicopy plasmids. Studying the phenotypes of the constructed yeast strains, we found differing abilities of particular human cornichon proteins to complement the functioning of yeast Erv14 homologue. Further, we studied the effect of the presence of human CNIH proteins on the plasma‐membrane targeting and activity of two alkali‐metal‐cation/H^+^ antiporters from the CPA (Cation/Proton Antiporter) family, a highly prevalent group of transmembrane transporters. While one of the tested transporters, yeast Nha1, belongs among transporters from the CPA1 subfamily of cation/proton antiporters, the other one, human Na^+^/H^+^ antiporter NHA2 (Fuster et al., 2008), is a member of the CPA2 transporter subfamily (Masrati et al., 2018). Our results show that the presence of human CNIHs supports the functioning of the yeast plasma‐membrane Na^+^, K^+^/H^+^ antiporter Nha1, and also improves the plasma‐membrane targeting and functioning of the human Na^+^/H^+^ antiporter NHA2 in yeast cells, which identifies NHA2 as a novel cargo of cornichon COPII cargo receptors.

## 2 RESULTS

### 2.1 Sequence and structure analysis of studied cornichon proteins

As mentioned above and summarized in (Papouskova, Cerna, et al., 2024; Papouskova, Zimmermannova, & Sychrova, 2024), the *S. cerevisiae* cornichon Erv14 (*Sc*Erv14) is 138 amino acids long (Figure 1a), and several motifs, identified experimentally, have been found to be important for its functioning (c.f. below). At first, we provided in this study a structural analysis of primary sequences and predicted 3D structures of yeast Erv14 and human CNIHs (Figure 1). Of the four human cornichon proteins, whose lengths vary from 139 to 160 amino‐acid residues (Figure 1a), CNIH2 and CNIH3 are the most similar (81.8% of identity, Figure 1b,c). On the other hand, CNIH4 proved to be the most distant from the human cornichons, sharing only approx. 32%–37% identity with CNIH1, CNIH2 and CNIH3. At the same time, CNIH4 is more related (44.4% identity) to the yeast cornichon Erv14 (Figure 1b,c). The overlaps of structural models of *Sc*Erv14 and human cornichons retrieved from the AlphaFold structural database (Figure 1d) show a high similarity of cornichons' structures, especially concerning the arrangement and mutual position of the four α‐helices. *Sc*Erv14 was found to be slightly prolonged in its C‐terminus when compared to human CNIH proteins (Figure 1a,d). The very end of *ScErv14's* C‐terminal part contains a conserved acidic motif, which has been identified in fungal and plant, but not human, cornichons. In *Sc*Erv14, this motif was suggested to be involved in the binding of the protein's cargoes (Rosas‐Santiago et al., 2017). A typical characteristic of CNIH2 and CNIH3 is the presence of a stretch of amino‐acid residues that is specific for these two cornichons and that is located at the beginning of their second α‐helix, which, consequently, is prolonged compared to the same part of *Sc*Erv14, CNIH1 or CNIH4 (Figure 1a,d).

**FIGURE 2:**
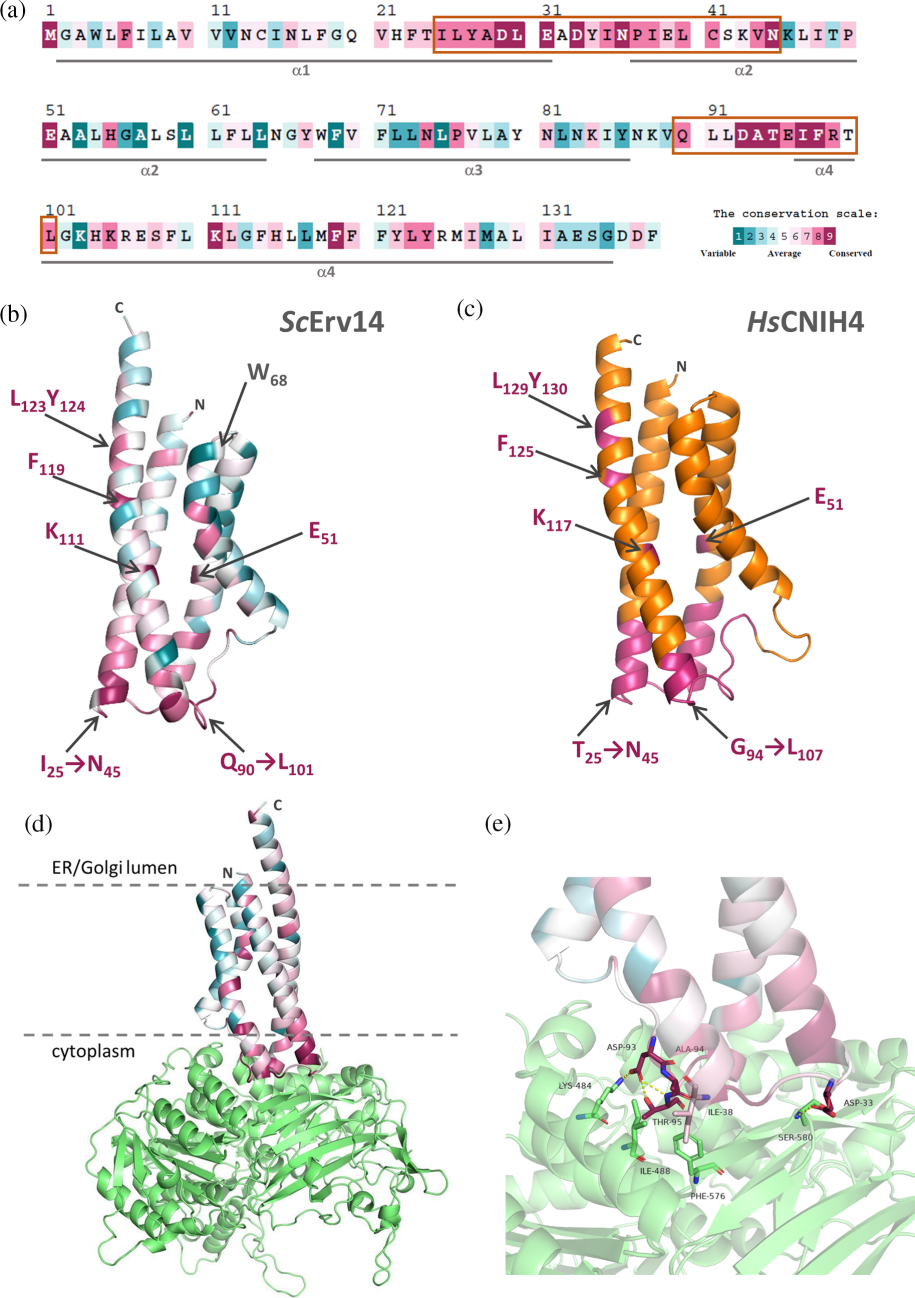
Evolutionary conservation of studied cornichons. (a) Evolutionary conservation analysis of *Sc*Erv14's amino‐acid residues. The colors correspond to the level of evolutionary conservation according to the presented scale. Gray lines show the positions of predicted α‐helical structures. Two stretches of highly conserved amino‐acid residues described in the text are highlighted by orange rectangles. (b, c) Structural model of *Sc*Erv14 and human CNIH4 (*Hs*CNIH4), respectively. In (b), the colors correspond to the level of evolutionary conservation according to (a). The position of the studied W68 and residues and regions with high evolutionary conservation scale described in detail in the text are highlighted. (d, e) Model of interaction of *S. cerevisiae* proteins Erv14 and Sec24. The model was created by using AlphaFold 3 and had a high level of confidence with both pTM and ipTM measures = 0.84 on the 0‐through‐1 scale. *Sc*Sec24 is colored in green. The colors in *Sc*Erv14 correspond to the level of evolutionary conservation. (d) Side‐view. The intrinsically disordered N‐terminal portion (aa 1–132) of *Sc*Sec24 is not shown. (e) A detailed view of putative interaction interface. The amino‐acid residues of *Sc*Erv14 D33, I38 and 93‐DAT‐95 (all of them being highly evolutionarily conserved) directly interact with *Sc*Sec24 via K484, I488, F576 and S580. Putative hydrogen bonds between highlighted amino‐acid residues are shown.

The second and third transmembrane domains of cornichon proteins create a “jack‐knife shape” with a tryptophan residue located at the junction and preserved in sequences of all four human CNIHs as well as *Sc*Erv14 (Figures 1a, 2b and S1a, b). This tryptophan was suggested to be a signature of all cornichon cargo receptors by (Nakagawa, 2019). However, neither mutation W68A nor W68P in *Sc*Erv14 changed the localization (Figure S1c) or functioning (Figure S1d) of its well‐characterized cargo—plasma‐membrane Na^+^, K^+^/H^+^ antiporter Nha1 (Rosas‐Santiago et al., 2016 and below). Hence, these results did not confirm the proposed necessity of this tryptophan for cornichons' structure/function.

To determine the critical residues in the cornichon COPII cargo receptors' family that can be important either for the binding to COPII‐coat protein complex or for the binding to a particular cargo, we next provided the evolutionary conservation (ConSurf) analysis based on an alignment of 1879 sequences (Figure S2). It revealed two longer stretches of highly conserved amino‐acid residues (with ConSurf score 8–9) as depicted in the sequence of yeast Erv14 (Figure 2a). A multiple sequence alignment of *Sc*Erv14 and human cornichons (Figure 1a) shows corresponding stretches of conserved residues in both yeast and human proteins. The first region with highly conserved residues, roughly ranging from I25 to N45 (in *Sc*Erv14), forms the end of the first α‐helix, the beginning of the second α‐helix and the short loop between them (Figure 2b,c). This stretch of amino‐acid residues also comprises the first part of a previously described DYPE (DY33‐34, PE50‐51) site of *Sc*Erv14 that was suggested to be involved in the incorporation of the cargo receptor into COPII vesicles (Pagant et al., 2015). E51 from the second part of the DYPE motif, which is predicted to be located in the middle of the second transmembrane domain, is also one of the most conserved amino‐acid residues (Figure 2b) and is preserved in all four human CNIHs (Figures 1a and 2c). The second identified stretch of conserved amino‐acid residues (Q90–L101) is predicted to be located in the loop connecting the third and fourth α‐helices and in the beginning of the fourth *Sc*Erv14's α‐helix. The region is partially formed by the *Sc*Erv14's amino‐acid residues 97‐IFRTL‐101, which were previously shown to play a role in the binding of the COPII‐coat protein complex by *Sc*Erv14 (D'Arcangelo et al., 2013; Pagant et al., 2015; Powers & Barlowe, 2002). The ConSurf analysis identified four more residues with the highest ConSurf score 8‐9‐K111 and F119 followed by LY123‐124 (in *Sc*Erv14 and fully preserved also in human CNIHs) that are located inside the fourth transmembrane domain of *Sc*Erv14, but their importance has not been fully characterized yet (Pagant et al., 2015).

In *S. cerevisiae*, Erv14 was suggested to be incorporated into COPII vesicles via its interaction with a so‐called D‐site (S491, F576, R578) of the Sec24 protein. In yeast, Sec24 forms a heterodimer with Sec23 and serves to bind COPII‐coated vesicle cargo proteins (D'Arcangelo et al., 2013; Pagant et al., 2015; Powers & Barlowe, 2002). The discovery of the abovementioned stretches of *Sc*Erv14's amino‐acid residues that show a significant tendency toward evolutionary conservation and at the same time comprise motifs that have been identified as being involved in the cornichon's packaging into COPII vesicles led us to the modeling and analysis of the putative interaction interface between *Sc*Sec24 and *Sc*Erv14 using AlphaFold 3. The resulting model (Figure 2d) had a high level of confidence, as indicated by the pTM and ipTM measures (Abramson et al., 2024), both of which assigned a value of 0.84 on the 0‐through‐1 scale. Both evolutionarily conserved regions of *Sc*Erv14 are indeed predicted to create the interaction interface with *Sc*Sec24. According to the model (Figure 2e), the *Sc*Erv14's amino‐acid residues D33, I38 and 93‐DAT‐95 (all of them being highly evolutionarily conserved, Figures 1a and 2a) directly interact with *Sc*Sec24 via hydrogen bonds or ionic and hydrophobic interactions. In *Sc*Sec24, the amino‐acid residues predicted to bind *Sc*Erv14 (K484, I488, F576 and S580) are either adjacent to or part of the D‐site of the protein. Thus, the model corresponds well to experimentally obtained data.

The role of evolutionarily conserved regions of cornichons in COPII‐coat protein complex binding was further supported by modeling the complexes of human cornichons CNIH1, CNIH2 and CNIH4 (proteins studied experimentally in our work) with human Sec24A protein. The complex of CNIH2 with Sec24A (and also the other three human Sec24 proteins) was predicted with confidence lower than 0.8 and therefore is not shown. Both identified evolutionarily conserved regions are suggested to be involved in Sec24A binding to CNIH1 as well as CNIH4 (Figure S3), similarly to what was identified for *Sc*Erv14 in interaction with *Sc*Sec24 (Figure 2e). Interestingly, all three models predict conserved D33 and the 93‐DAT‐95 motif of *Sc*Erv14 and corresponding residues in human cornichons to be in contact with Sec24 proteins. Together, our sequence analysis and protein interaction modeling show that the most conserved parts of cornichon proteins bind with high probability to the coat protein complex of COPII vesicles.

### 2.2 Human cornichons partially complement the Erv14's functions in the maintenance of cation homeostasis in *S. cerevisiae* cells

To evaluate the possibility of using yeast cells to study the properties of human cornichons, we purchased plasmids harboring cDNAs encoding human CNIH1, CNIH2, and CNIH4 (CNIH3 was omitted from this study due to its high similarity to CNIH2, as shown in Figure 1b,c) with codons optimized for expression in *S. cerevisiae* (Figure S4). Using these cDNAs, we prepared (i) YEplac181‐based plasmids (Table S1) in which the corresponding cDNAs were tagged with the hemagglutinin‐encoding sequence (HA) and (ii) BYT45‐derived strains (Table 1) in which the cDNAs were inserted into the genomes, replacing the coding sequence of *ScERV14* gene. The unique BYT45 strain is highly sensitive to increased concentration of Na^+^ (or its toxic analogue Li^+^) due to the lack of genes encoding alkali‐metal‐cation exporters (Na^+^, K^+^‐ATPases Ena and Na^+^, K^+^/H^+^ antiporter Nha1) (Navarrete et al., 2010). We used this strain because, in our previous studies, we characterized BYT45 strain and its *erv14Δ* derivative (Table 1) to be suitable for studying the role of yeast and plant cornichons in the proper trafficking of alkali‐metal‐cation transporters (Papouskova et al., 2021;Rosas‐Santiago et al., 2016; Zimmermannova et al., 2019). With the advantage of overexpressing genes from multicopy plasmids, we first assessed the ability of yeast cells to produce human cornichons by preparing total protein extracts from BYT45*erv14Δ* cells transformed with multi‐copy plasmids encoding HA‐tagged yeast or human cornichons (Table S1). The production of cornichons was detected using an anti‐HA antibody. As Figure S5a shows, though all the studied cornichon ORFs (encoding *Sc*Erv14 and three human CNIHs) were expressed in yeast cells from the same multi‐copy plasmids under the control of the same promoter (of the *ScERV14* gene), the amount of detected human proteins was clearly decreased when compared to *Sc*Erv14. This result might suggest a lower stability of human cornichons heterologously expressed in *S. cerevisiae* cells.

**TABLE 1:** Strains used in this study.

Strain	Genotype	Source/reference
BY4741	*MAT*a *his3Δ1 leu2Δ0 met15Δ0 ura3Δ0*	EUROSCARF
BY4741^pHl^	BY4741 *his3Δ1::loxP‐kanMX‐loxP‐GPD1* ^*P*^ *‐pHluorin*	(Zimmermannova et al., 2015)
BY4741*erv14Δ*	BY4741 *erv14Δ::loxP*	(Rosas‐Santiago et al., 2015)
BY4741*erv14Δ* ^pHl^	BY4741 *erv14Δ his3Δ1::loxP‐kanMX‐loxP‐GPD1* ^*P*^ *‐pHluorin*	(Zimmermannova et al., 2019)
BYT45	BY4741 *nha1Δ::loxP ena1‐5Δ::loxP*	(Navarrete et al., 2010)
BYT45*erv14Δ*	BYT45 *erv14Δ::loxP*	(Rosas‐Santiago et al., 2015)
BYT45‐CNIH1	BYT45 *erv14Δ::HsCNIH1‐TPS1* ^*T*^ *‐loxP‐kanMX‐loxP*	This study
BYT45‐CNIH2	BYT45 *erv14Δ::HsCNIH2‐TPS1* ^*T*^ *‐loxP‐kanMX‐loxP*	This study
BYT45‐CNIH4	BYT45 *erv14Δ::HsCNIH4‐TPS1* ^*T*^ *‐loxP‐kanMX‐loxP*	This study

**FIGURE 3:**
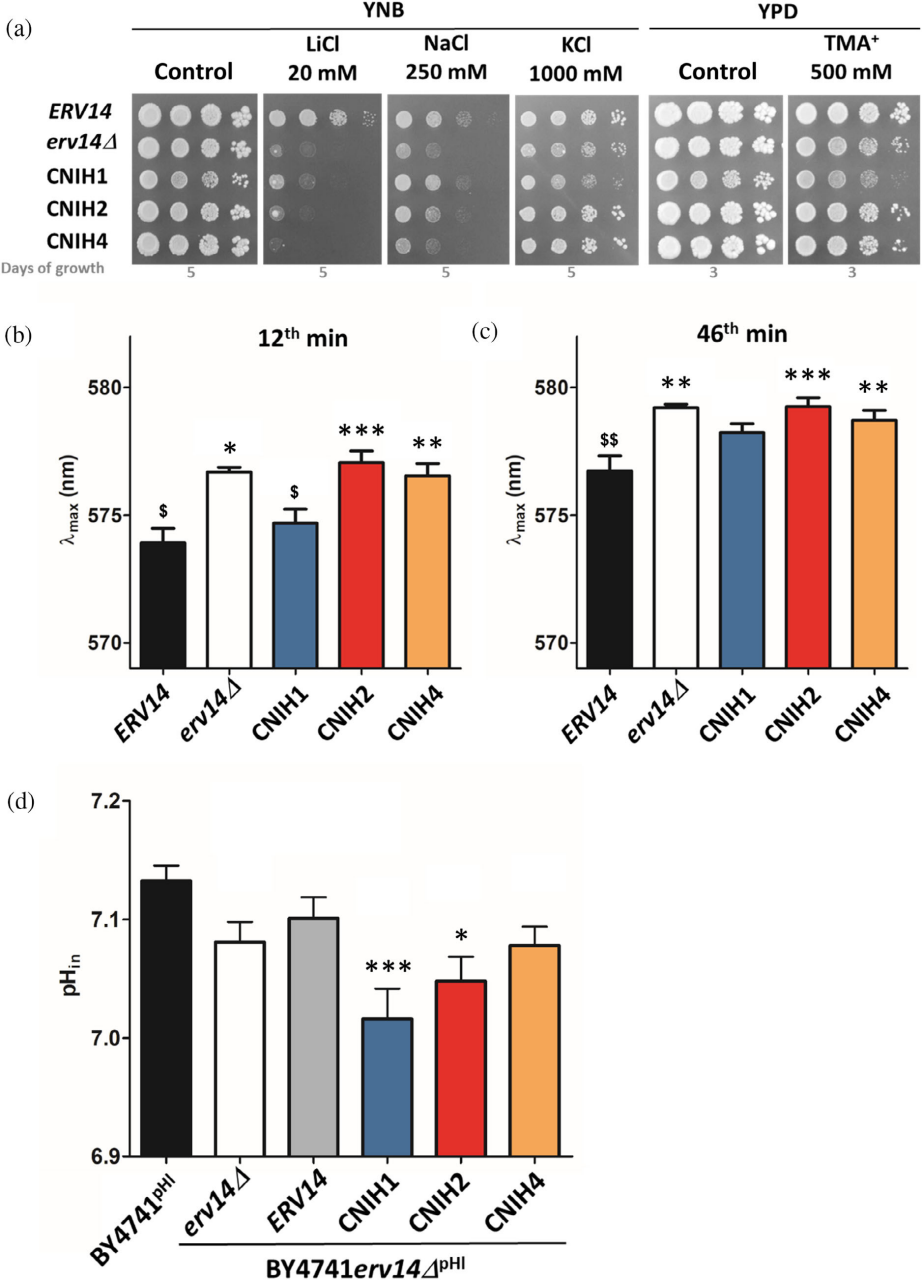
Characterization of *S. cerevisiae* strains that produce human cornichons. (a) Growth of BYT45 (*nha1Δ ena1‐5Δ*) cells with *ERV14* or without the gene (*erv14Δ*) or strains with *ERV14* replaced with human CNIH cDNAs in the genome on plates supplemented with salts or TMA^+^ as indicated. Results of a representative experiment of at least three independent repetitions are shown. (b, c) Relative membrane potential of the same strains as in (a) shown as the mean λ_max_ value reached at 12 min (b) or 46 min (c) after the addition of the diS‐C_3_(3) fluorescent probe. Data represent the mean values ± SEM from four independent experiments (with two technical replicates each). (d) Intracellular pH of BY4741^pHl^ cells with or without the *ERV14* gene (BY4741*erv14Δ* ^pHl^) that produce pHluorin. Strains were transformed either with an empty plasmid or with plasmids encoding various cornichons as indicated. Data represent the mean values ± SEM from eight independent experiments (with eight technical replicates each). Symbols * or ^$^ in (b–d) indicate statistically significant differences to control cells with *ERV14* (black column) or without *erv14Δ* (white column), respectively, */^$^ *p* < 0.05, **/^$$^ *p* < 0.01, ***/^$$$^ *p* < 0.001.

In BYT45 cells, the deletion of *ERV14* gene results in various phenotypes connected to alkali‐metal‐cation homeostasis, such as the worsening of growth in the presence of high Na^+^, Li^+^, K^+^, or cationic drug tetramethylammonium (TMA^+^) concentrations, the relative membrane hyperpolarization, or drop in intracellular pH (Rosas‐Santiago et al., 2016; Zimmermannova et al., 2019). To examine whether the studied human cornichons are functional in *S. cerevisiae*, we compared these physiological parameters of BYT45 (with *ERV14*) and BYT45*erv14Δ* cells with strains in which the *ERV14* coding region was replaced with human cDNAs encoding CNIH1, CNIH2, or CNIH4 (Table 1 and Figure 3). Integration of human coding sequences into the genome behind the *ERV14* promoter was chosen to ensure an expression profile typical for the host's own cornichon cargo receptor. First, we tested the tolerance of cells to salts and cationic TMA^+^ by monitoring the growth of these five strains on plates supplemented with salts (NaCl, LiCl, KCl) or TMA^+^ (Figure 3a). The production of CNIH1, but not the other human cornichons, was somehow toxic, which could be observed as a worse growth of cells with CNIH1 on control plates (without salts or TMA^+^) compared to the other strains (Figure 3a). On the other hand, irrespective of this negative impact on cell growth, the presence of CNIH1 partially complemented the decreased LiCl and NaCl (but not KCl or TMA^+^) tolerance of BYT45*erv14Δ* cells (Figure 3a). A less pronounced increase in tolerance to the presence of Na^+^ was also observed for cells producing CNIH2. In contrast to the strain with CNIH1, cells producing CNIH2 or CNIH4 improved the growth of cells in the presence of KCl and TMA^+^ (Figure 3a). The presence of CNIH4 had no effect on the ability of cells to cope with NaCl and slightly decreased the growth of cells in the presence of highly toxic LiCl (Figure 3a). The same phenotypes observed on control media and on NaCl and LiCl were also confirmed when the growth of these strains was monitored in liquid media (Figure S6a). Interestingly, the expression of CNIH1 cDNA from a multi‐copy plasmid also partially complemented the function of Erv14 in NaCl tolerance of wild‐type cells BY4741, which contain alkali‐metal‐cation exporters (Figure S5b).

We have previously shown that the lack of the *ERV14* gene resulted in a relative plasma‐membrane hyperpolarization of the wild‐type as well as BYT45 cells (Zimmermannova et al., 2019, and also Figure 3b,c and S6b). To test if human cornichons can substitute *Sc*Erv14's role in the maintenance of plasma‐membrane potential, we compared the relative plasma‐membrane potential of the same five strains as above. Cells pre‐grown in YPD to the mid logarithmic phase were stained by diS‐C_3_(3) fluorescent probe as described previously (Zimmermannova et al., 2019). Comparison of staining curves is shown in Figure S6b. Mean values of λ_max_ for each strain reached after 12 and 46 min are shown in Figure 3b,c.

Of all three human cornichons, only the presence of CNIH1 was able to partially decrease the relative hyperpolarization of cells caused by the *erv14* deletion. Shortly after the addition of the probe, the maximum emission wavelength (λ_max_ determined at 12^th^ min of staining, Figure 3b) detected for cells producing CNIH1 was not significantly different from the value obtained for cells with Erv14, whereas the λ_max_ values at 12^th^ min measured for the other strains were higher (Figures 3b and S6b). However, with increased staining time, a relative hyperpolarization of cells with CNIH1 compared to the strain with *ERV14* became obvious, and λ_max_ values at 46 min of staining detected for cells with CNIH1 came closer to the values measured for cells without Erv14 or producing CNIH2 or CNIH4 (Figures 3c and S6b).

By using a pH‐sensitive GFP variant pHluorin (Miesenbock et al., 1998), we previously showed that the deletion of *ERV14* in BY4741 cells results in a decrease in intracellular pH (pH_in_) (Zimmermannova et al., 2019). Therefore, we next tested whether the expression of human cornichons in yeast cells can influence the cytosolic pH level. For this experiment, we used BY4741*erv14Δ* ^pHl^ strain, chromosomally expressing pHluorin (Table 1) and containing either the empty vector YEplac181 or multi‐copy YEplac181‐based plasmids for the expression of either *ScERV14* or human CNIH1, CNIH2 or CNIH4 cDNAs under the control of the *Sc*Erv14 promoter (Table S1). Cells were cultivated in a low‐fluorescent YNB medium to the exponential phase and determined pH_in_ values were compared to the wild‐type strain BY4741^pHl^ expressing pHluorin and containing the empty vector. In agreement with previously published results (Zimmermannova et al., 2019), the pH_in_ level was lower in BY4741*erv14Δ* cells than in the wild‐type strain (Figure 3d), and the production of Erv14 was able to increase the pH_in_ drop in BY4741*erv14Δ* cells. However, the presence of neither human cornichon was able to complement the decrease in pH_in_ caused by *erv14* deletion (Figure 3d). In cells with CNIH1, we detected even lower pH_in_ when compared to cells without Erv14 (Figure 3d). This finding indicates a highly disturbed H^+^ homeostasis in cells with this particular human cornichon, which might result in a slower growth of cells even under control conditions (Figures 3a and S6a).

Taken together, characterization of strains producing human cornichons CNIH1, CNIH2 or CNIH4 revealed that each paralog can complement some of the pleiotropic Erv14's functions in the maintenance of cation homeostasis differently (and only partially). This demonstrates a possible divergence of functions/cargoes of each cornichon protein in mammalian cells.

### 2.3 Human cornichons CNIH1 and CNIH4 improve the functioning of *S. cerevisiae* Na^+^, K^+^/H^+^ antiporter Nha1 in yeasts

**FIGURE 4:**
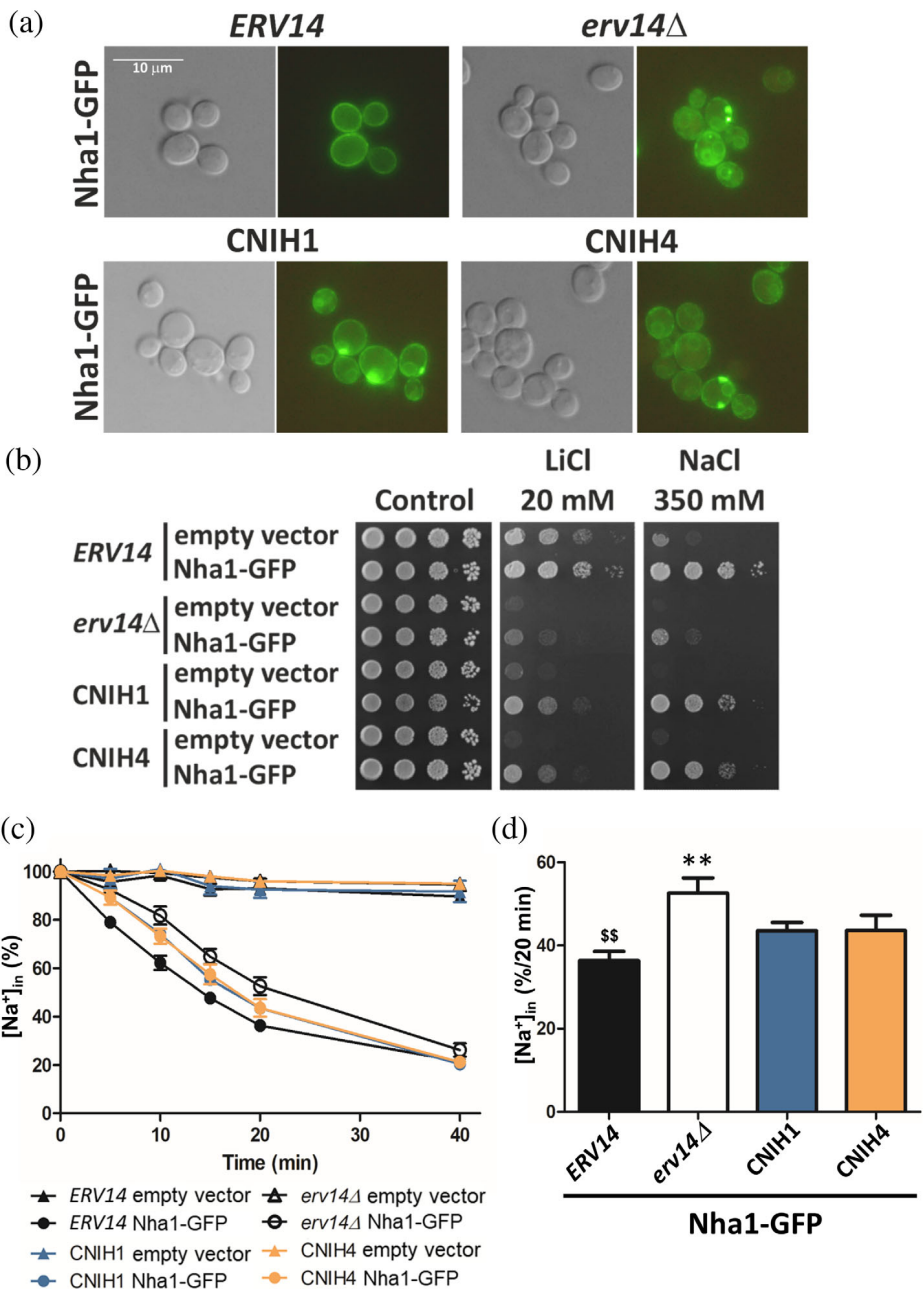
The influence of human cornichons on the localization and functioning of Na^+^, K^+^/H^+^ antiporter *Sc*Nha1 in *S. cerevisiae* cells. (a) Localization of *Sc*Nha1‐GFP in BYT45 (*nha1Δ ena1‐5Δ*) cells with *ERV14* or without the gene (*erv14Δ*) or strains with *ERV14* replaced by human CNIH1 or CNIH4 cDNAs in the genome as indicated. Transformants were observed under a fluorescence microscope (right), and a Nomarski prism was used for whole‐cell imaging (left). (b) Growth of *Sc*Nha1‐GFP‐producing BYT45 derivatives as in (a) and the same strains containing the empty vector on plates supplemented with salts as indicated. The pictures were taken after 4 days of growth. Results of a representative experiment of at least three independent repetitions are shown. (c, d) Na^+^ loss from *Sc*Nha1‐GFP‐producing BYT45 cells as in (a). Results are expressed as % of the value measured at time 0. The average initial amount of Na^+^ in cells was 109.24 ± 6.84 nmol/mg dry weight. (c) Entire Na^+^ efflux curves. (d) Na^+^ content in cells after 20 min of measurements of Na^+^ efflux. Data shown represents mean values ± SEM of at least three independent measurements. Symbols * or ^$^ indicate statistically significant differences to control cells with *ERV14* (black column) or without *erv14Δ* (white column), respectively, **/^$$^ *p* < 0.01.

To further evaluate the possibility of studying the functioning and properties of human cornichons in yeast cells, we studied their ability to support the plasma‐membrane localization and cation‐export activity of the yeast Na^+^, K^+^/H^+^ antiporter Nha1, one of the known cargoes of the *S. cerevisiae* cornichon Erv14 (Herzig et al., 2012; Papouskova et al., 2021; Rosas‐Santiago et al., 2016). For these experiments, we used BYT45 derivatives with either CNIH1 or CNIH4 cDNAs integrated into the genome (Table 1). CNIH1 was chosen due to its ability to partially substitute Erv14 function in cell tolerance to NaCl or LiCl in BYT45 (Figure 3a) as well as in BY4741 strain that possesses Nha1 (Figure S5b). CNIH4 was tested as it shares the highest % identity with *Sc*Erv14 of all the studied human CNIHs (Figure 1c). The BYT45 derivatives producing human cornichons (and lacking the chromosomal copy of *NHA1* gene) were transformed with a multi‐copy plasmid encoding Nha1 tagged with GFP at the hydrophilic C‐terminus (Table S1). As shown in Figure 4a, Nha1‐GFP was properly localized to the plasma membrane in cells with *ERV14*, while in *erv14Δ* cells, the antiporter was partially accumulated intracellularly in the ER. A partial ER stacking of Nha1 could also be observed in cells with either CNIH1 or CNIH4 (Figure 4a). To quantitatively evaluate the degree of observed Nha1‐GFP stacking in ER in particular transformants, we analyzed 20 of each type of cells and quantified the levels of corrected total fluorescence in the perinuclear ER (CTCF_pnER_) and in the surface area (CTCF_surface_) in individual cells and calculated the ratios of the obtained values. A higher CTCF_pnER_/CTCF_surface_ ratio means a higher degree of ER stacking in perinuclear ER. The calculated CTCF_pnER_/CTCF_surface_ value was slightly lower for cells producing CNIH4 than for *erv14Δ* cells; however, the difference was not statistically significant (Figure S7).

The structure of cortical ER is complex in yeast cells, with the extent of contact between ER and plasma membrane (West et al., 2011). In cells with CNIH1 or CNIH4, we observed a more continuous fluorescence signal in the cell periphery compared to *erv14Δ* cells (Figure 4a), which might indicate an improved plasma‐membrane versus cortical ER localization of Nha1‐GFP in cells with human cornichons than in *erv14Δ* cells. In our previous publications, we showed that besides microscopy, the role of cornichon cargo receptors in plasma membrane targeting can be assessed functionally by measuring the activity of their associated transporters (Papouskova et al., 2021; Rosas‐Santiago et al., 2016; Zimmermannova et al., 2019). Yeast Nha1 antiporter, as a typical secondary active transporter, is able to export all monovalent cations (Na^+^, Li^+^, K^+^, Rb^+^) from cells in exchange for protons (Kinclova et al., 2001). Thus, next, we estimated the activity of *Sc*Nha1 in the same set of strains as above by (i) growth tests in the presence of NaCl or LiCl and (ii) by direct measurements of *Sc*Nha1‐mediated Na^+^ efflux from cells (Figure 4b,c). Both experiments demonstrate that the presence of human CNIH1 or CNIH4 improved the functioning of *Sc*Nha1 antiporter, though not as efficiently as the yeast Erv14. The drop test shown in Figure 4b shows that cells with *Sc*Nha1 and producing CNIH1 or CNIH4 clearly coped better with the presence of salts than *erv14Δ* cells, but not as well as cells with *Sc*Nha1 and *Sc*Erv14. Measurements of Na^+^‐export activity of *Sc*Nha1 from cells with different cornichons fully confirmed our results from drop test experiments. Due to a decreased amount of *Sc*Nha1 in the plasma membrane, sodium loss from *erv14Δ* cells was significantly lower than from cells with *Sc*Erv14 (Figure 4c,d). The presence of human cornichons increased the *Sc*Nha1‐mediated Na^+^ export from cells, although the amount of Na^+^ lost from these cells did not reach the level observed in cells with *Sc*Erv14 (Figure 4c,d).

Therefore, examining the level of *Sc*Nha1's ability to export Na^+^ (or Li^+^) from cells confirmed that human CNIH1 and CNIH4 can partially compensate for the lack of *Sc*Erv14 and assist *Sc*Nha1 in reaching the plasma membrane in yeast cells (Figure 4). At the same time, these experiments demonstrate that the assays evaluating the functioning of *Sc*Nha1 in individual strains are more sensitive and suitable for assessing the degree of plasma‐membrane targeting of the antiporter (which is a prerequisite of its proper functioning) than observations of the localization of the GFP‐tagged transporter.

### 2.4 Both *S. cerevisiae* Erv14 and human cornichons improve the plasma‐membrane localization and functioning of the human Na^+^/H^+^ antiporter NHA2 in yeast cells

**FIGURE 5:**
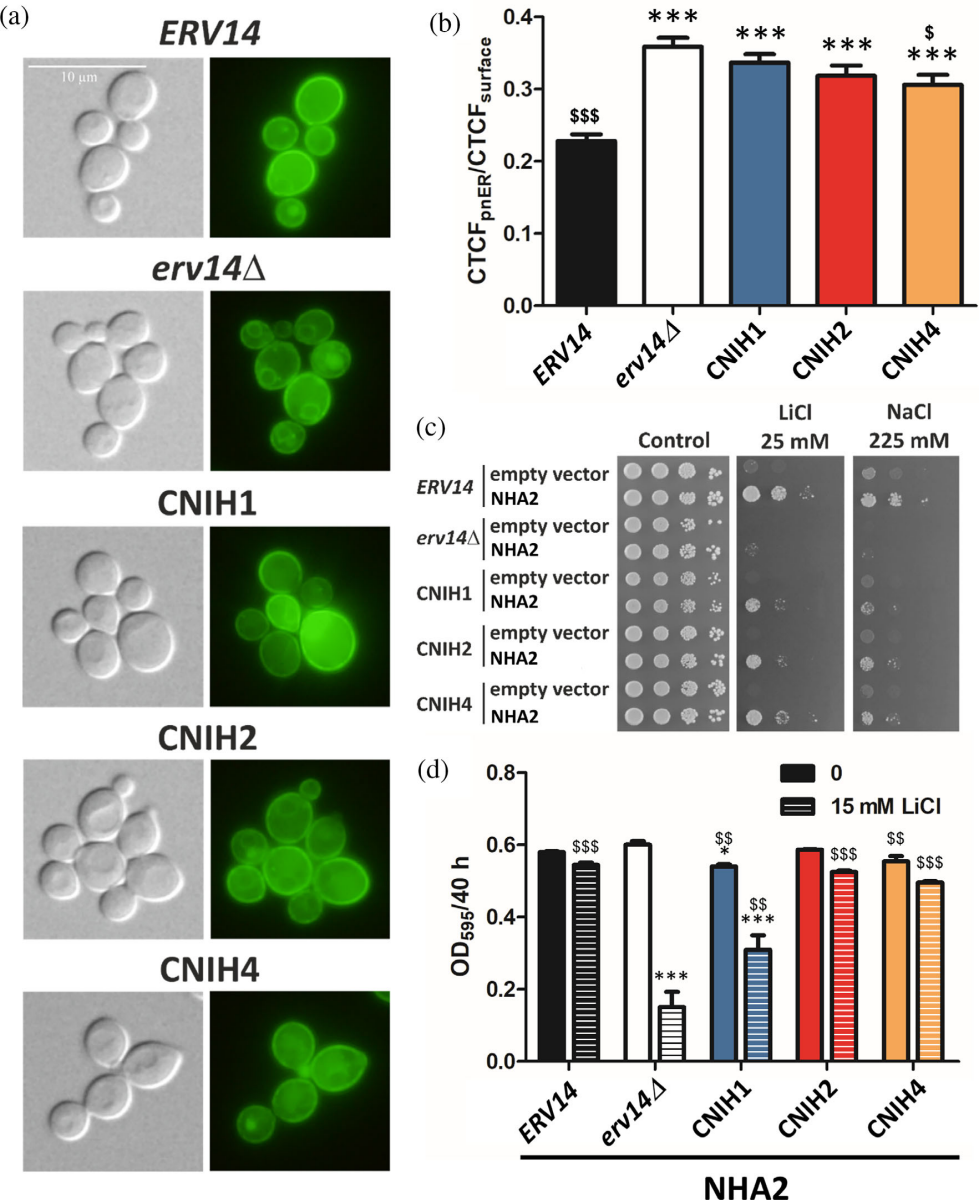
The influence of human cornichons on the localization and functioning of human Na^+^/H^+^ antiporter NHA2 in *S. cerevisiae* cells. (a) Localization of GFP‐NHA2 in BYT45 (*nha1Δ ena1‐5Δ*) cells with *ERV14* or without the gene (*erv14Δ*) or strains with *ERV14* replaced with human CNIH cDNAs in the genome. Transformants were observed under a fluorescence microscope (right), and a Nomarski prism was used for whole‐cell imaging (left). (b) The level of GFP‐NHA2 perinuclear ER (pnER) accumulation in strains shown in (a) expressed as the ratios of CTCF_pnER_/CTCF_surface_. The fluorescence signal in 20 cells was analyzed for each strain. Data represent mean values ± SEM. (c) Growth of BYT45 derivatives as in (a) expressing the NHA2 from a multi‐copy plasmid and the same strains containing the empty vector on plates supplemented with salts as indicated. The pictures were taken after 4 days of growth. Results of a representative experiment of at least three independent repetitions are shown. (d) Optical density of NHA2‐producing BYT45 cells as in (c) cultivated for 40 h in liquid media supplemented with LiCl as indicated. Data represents the mean values ± SEM of three independent measurements. Symbols * or ^$^ in (b) and (d) indicate statistically significant differences to control cells with *ERV14* (black column) or without *erv14Δ* (white column), respectively, */^$^ *p* < 0.05, **/^$$^ *p* < 0.01, ***/^$$$^ *p* < 0.001 (in (d) in the corresponding medium).

In the final part of our study, we examined whether the plasma‐membrane targeting of the human Na^+^/H^+^ antiporter NHA2 is dependent on the presence of cornichons in yeast cells. NHA2 is functional in *S. cerevisiae* cells, where it is targeted to the plasma membrane and exports Na^+^ and Li^+^ cations (Velazquez et al., 2022). First, we transformed BYT45, BYT45*erv14Δ* or BYT45 derivatives with integrated cDNAs of human CNIH1, CNIH2 or CNIH4 instead of *ERV14* (Table 1) with a plasmid encoding human NHA2 N‐terminally tagged with GFP (Table S1), and studied the localization of the antiporter. In cells with *Sc*Erv14, GFP‐NHA2 was predominantly targeted to the plasma membrane, though in some cells, a weak GFP signal was also observed inside cells (Figure 5a). However, in cells without Erv14, we observed a highly increased accumulation of GFP‐NHA2 in the perinuclear ER, which strongly suggested that the transporter's trafficking through the secretory pathway depends on the presence of yeast Erv14 in cells (Figure 5a). Moreover, NHA2's plasma‐membrane localization also seemed to be improved in cells producing human cornichons instead of *Sc*Erv14 (Figure 5a). Quantification of fluorescence and the degree of ER stacking of human GFP‐NHA2 in perinuclear ER (by calculation of CTCF_pnER_/CTCF_surface_ ratios) revealed that the presence of *Sc*Erv14 in cells significantly decreased the CTCF_pnER_/CTCF_surface_ value when compared with the same ratio counted for *erv14Δ* cells, which indicates the improved plasma‐membrane targeting of GFP‐NHA2 in cells with *Sc*Erv14 (Figure 5b). In comparison with *erv14Δ* cells, the presence of human cornichons CNIH1 or CNIH2 also decreased the CTCF_pnER_/CTCF_surface_ ratio of fluorescence and it was significantly lower in cells producing CNIH4 and GFP‐NHA2. It clearly demonstrates the ability of this human cornichon to help NHA2 to reach the plasma membrane in yeast cells (Figure 5b).

To confirm this, we further assessed the effect of cornichon proteins on the functioning of NHA2 by growth tests and cation efflux measurements. The fusion with GFP at the N‐terminus of NHA2 was shown to influence its ability to export Na^+^ and Li^+^ from cells (Velazquez et al., 2022). Therefore, we prepared a new set of BYT45 derivatives (without Erv14 or with yeast or human cornichons) expressing NHA2 without the GFP tag from a multi‐copy plasmid (Table S1), and tested their growth on plates supplemented with LiCl or NaCl. As shown in Figure 5c, NHA2 better supported the growth of cells on salts when any of the studied cornichons was present in cells, although cells producing NHA2 and the yeast Erv14 exhibited the highest salt tolerance. To confirm the improvement of NHA2's activity in the presence of *Sc*Erv14 or human CNIHs, we measured Na^+^ loss from cells producing NHA2. When compared to *erv14Δ* cells, a significantly higher Na^+^ efflux activity of NHA2 was detected only in cells producing NHA2 and the yeast cornichon Erv14 (Figure S8a). It is worth noting here that the cation‐export activity of NHA2 is considerably lower in yeast cells than the activity of *S. cerevisiae*'s own Na^+^, K^+^/H^+^ antiporter Nha1 (cf. the loss of more than 60% of Na^+^ in BYT45 with *Sc*Nha1 in 20 min vs. the loss of ≈25% of Na^+^ in the same cells with human NHA2 in 60 min of measurement, Figures 4d and S8a). The relatively low Na^+^ export activity of NHA2 might impede the detection of the positive effect of human cornichons on the functioning of NHA2 in these short‐term cation‐export measurements. Therefore, in the last experiment, we examined the ability of various cornichons to support the functioning of NHA2 in long‐term growth in liquid media without or with the addition of LiCl (Figures 5d and S8b). Interestingly, in control medium (without LiCl), strains with the human CNIH2 or CNIH4 grew better than *erv14Δ* cells, which indicates the ability of CNIH2 or CNIH4 to complement the growth defect caused by the lack of *Sc*Erv14 (Figure S8b). The slowest growth in control medium was observed for cells producing the CNIH1 (Figure S8b), which is in full agreement with experiments shown in Figures 3a and S5a and proves a negative effect of CNIH1 on cell growth. In the presence of LiCl, all strains with NHA2 and either of the studied cornichons grew better than *erv14Δ* cells with NHA2 (Figures 5d and S8b).

In summary, these results demonstrate that cornichon cargo receptors are crucial for the efficient plasma membrane targeting and functioning of human NHA2. When produced in yeast cells, the CNIH4 paralogue was the most efficient among the human cornichons at supporting the NHA2 plasma‐membrane localization together with its transport activity. However, the apparently more sensitive functional assays (see above) indirectly revealed that all tested human cornichons were able to support NHA2's plasma membrane targeting in *S. cerevisiae* cells. Although it is necessary to confirm the physical interaction between NHA2 and human cornichons directly in human cells, our results strongly suggest NHA2 to be a new interaction partner of cornichon COPII‐coated vesicle cargo receptors.

## 3 DISCUSSION

Cation transporters are typically large polytopic membrane proteins with hydrophilic parts located on both sides of the membrane. Besides proper folding and maturation, an essential prerequisite of transporters' functioning is their trafficking to the target membrane, where the proteins fulfill their physiological functions. This study advances our understanding of cornichon COPII‐cargo receptors, which facilitate the efficient transport of diverse membrane proteins, by (i) revealing novel structural and functional features of *S. cerevisiae* Erv14 and human CNIH1‐4 and (ii) introducing a yeast‐based expression system designed to identify new Erv14/CNIH cargoes. Using this system, we successfully demonstrated human Na^+^/H^+^ antiporter as a novel cargo of cornichon cargo receptors.

In *S. cerevisiae*, the incorporation of Erv14 into COPII vesicles is mediated by its interaction with Sec24, a cargo binding adaptor and component of the Sec23/Sec24 heterodimer (D'Arcangelo et al., 2013; Pagant et al., 2015; Powers & Barlowe, 2002). Whereas the cargo repertoire of individual cornichons might have diverged during evolution, the ability to interact with COPII‐complex proteins is absolutely crucial for cornichons' functioning as COPII cargo receptors. In agreement, our ConSurf analysis based on a large alignment of cornichons (1879 sequences) from fungal, plant and animal species (Figure S2) and AlphaFold3 modeling of yeast Erv14 or human CNIH1 or CNIH4 in complex with yeast or human Sec24 proteins confirmed that corresponding contact sites are within cornichons' most evolutionarily conserved amino‐acid regions (Figures 2d,e and S3). In full accord, the evolutionarily most conserved regions also contain few amino‐acid residues that were previously experimentally shown in *Sc*Erv14 to be involved in the interaction of the cargo receptor with the COPII‐coat complex (Pagant et al., 2015; Powers & Barlowe, 2002).

So far, only little information is known as concerns contact sites between cornichon proteins and their cargoes. In *Sc*Erv14, the FLN motif (FL62‐63, N74) has a proposed role in the binding of at least some of the receptor's cargo proteins (Pagant et al., 2015). These amino acids are presumably buried in the membrane as part of the second and third transmembrane domains, and only N74 is among the highest evolutionarily conserved amino‐acid residues (Figure 2a). The acidic amino‐acid residues from the C‐terminal part (E133, DD136‐137) are also thought to be involved in the binding of cargoes by *Sc*Erv14 (Rosas‐Santiago et al., 2017). However, of these amino‐acid residues only D137 exhibits some tendency toward evolutionary conservation (Figure 2a). The interaction interface between transmembrane domains of mammalian cornichons CNIH2/3 and AMPA receptors, their only known cargoes so far, involves amino‐acid residues located in the first and second α‐helices of the cornichon proteins (Gangwar et al., 2023; Nakagawa, 2019; Yu et al., 2021; Zhang et al., 2021). Whether some other of the newly identified evolutionarily conserved amino‐acid residues located inside the membrane are involved in the recognition of cargo proteins by cornichon cargo receptors remains to be elucidated.

Several pieces of experimental evidence suggest that the way that cornichons recognize not only COPII‐complex components but also cargo proteins might be at least partially evolutionarily conserved. (i) Plant (*Oryza sativa* CNIH1 and *Arabidopsis thaliana* CNIH1, CNIH3 and CNIH4) cornichons are able to support the trafficking of Erv14's cargoes in yeast *erv14Δ* cells, (ii) *Sc*Erv14 helps the rice cation transporter *Os*HKT1;3 to exit the ER in yeast cells and (iii) human CNIH1 can complement the non‐axial budding phenotype of *S. cerevisiae* cells that lack Erv14 (Castro et al., 2007; Rosas‐Santiago et al., 2015; Rosas‐Santiago et al., 2017; Wudick et al., 2018). Our study confirms the use of yeast cells, which can be both easily cultivated and genetically manipulated, to be of benefit for obtaining more information about the functioning and cargo repertoires of human cornichons CNIH1, CNIH2 and CNIH4. Characterization of yeast strains producing individual human cornichons (Figures 3, S5b, and S6) demonstrates that their ability to complement the *Sc*Erv14's functions connected to the cation homeostasis (cell salt and TMA^+^ tolerance, membrane potential, intracellular pH) depends on the presence of particular CNIH paralog. At the same time, the extent to which individual CNIH proteins were able to substitute for Erv14's function also varied. These results clearly show an evolutionary divergence of functions and possibly cargo repertoires of tested human cornichons.

The most significant phenotypes were observed for cells expressing the CNIH1. Despite its production slowed growth of cells (Figure 3a and S6a) and caused a drop in intracellular pH (Figure 3d), CNIH1 was the most efficient at supporting the growth of *S. cerevisiae erv14Δ* cells in the presence of toxic Na^+^ and Li^+^ cations (Figure 3a). The increased Na^+^ and Li^+^ tolerance of cells with CNIH1 might be the consequence of relative cell depolarization (Navarrete et al., 2010), as CNIH1 was also the only human cornichon capable of transiently decreasing the plasma‐membrane potential of cells in comparison with the relatively hyperpolarized cells lacking Erv14 (Figure 3b,c and S6b). Two K^+^‐specific transporters, whose activity influences cell plasma‐membrane potential, the high‐affinity K^+^ importer Trk1 and voltage‐gated channel Tok1, are among Erv14's cargoes (Madrid et al., 1998; Maresova et al., 2006; Navarrete et al., 2010; Zimmermannova et al., 2019). Our preliminary results show that the production of human CNIH1 can improve the plasma‐membrane targeting of Tok1 in *S. cerevisiae* cells (our unpublished results summarized in Figure S9). This finding demonstrates the usefulness of our heterologous expression system for the identification of new *Sc*Erv14/CNIH cargoes and suggests that CNIH1 can be an important cargo receptor for K^+^ channels. Therefore, we are currently using our yeast‐based expression system for testing various human K^+^ channels as putative cargoes of human CNIHs.

CNIH2 and CNIH4 were able to partially substitute *Sc*Erv14 in supporting cell tolerance to TMA^+^, despite their inability to decrease cell plasma‐membrane potential (Figure 3 and S6b). Several yeast multidrug transporters (Pdr5, Pdr12, Qdr2, Yor1) have been identified as cargoes of *Sc*Erv14 (Herzig et al., 2012; Louie et al., 2012; Pagant et al., 2015; Rosas‐Santiago et al., 2017). Whether human CNIH2 or CNIH4 interacts with and improves the plasma‐membrane targeting of any of these proteins, which might consequently result in an increased ability of cells to cope with high TMA^+^ levels in medium, remains to be discovered.

Studying the localization and functioning of GFP‐tagged *Sc*Nha1, a known cargo of *Sc*Erv14 (Herzig et al., 2012; Rosas‐Santiago et al., 2016), in cells producing human cornichons CNIH1 or CNIH4 instead of *Sc*Erv14 (Figures 4 and S7) did not clearly show an effect of the production of human CNIHs on the plasma‐membrane targeting of the yeast antiporter, but revealed that both human cornichons were able to support the functioning of Nha1 (Figure 4b–d). Human cornichons improved Nha1's functioning to a lesser degree than *S. cerevisiae*'s own cornichon Erv14, which might be the consequence of several factors. (i) As shown in Figure S5a, lower amounts of human cornichons were detected in yeast cells compared to the amount of *Sc*Erv14. (ii) The interactions of heterologously expressed human CNIHs with either yeast COPII‐coat protein complex or Nha1 itself might be weaker. In contrast to CNIHs, *Sc*Erv14 is prolonged at its C‐terminus (Figures 1a,d and S1a), and these C‐terminal amino‐acid residues were suggested to be involved both in binding the cargoes by *Sc*Erv14 (E133, D136, D137) and packaging of the cornichon into COPII‐coated vesicles (S134) (Lagunas‐Gomez et al., 2023; Rosas‐Santiago et al., 2017). (iii) Besides the simple plasma‐membrane targeting of Nha1, *Sc*Erv14 was also shown to be necessary for the antiporter's oligomerization, which seems to take place in the ER of cells (Mitsui et al., 2005; Rosas‐Santiago et al., 2016). We have previously shown that deletion of *ScERV14* resulted in a decrease of dimer/monomer ratio of Nha1 in the ER, and solely the monomeric form was detected in the plasma membrane of cells (Rosas‐Santiago et al., 2016). Whether human cornichons are able to efficiently support the dimerization of *S. cerevisiae* Nha1 in the membrane of ER is not known at this moment. Unfortunately, AlphaFold 3 modeling of Nha1 (either as a monomer or as a dimer) in complex with *Sc*Erv14 or human cornichons by AlphaFold3 did not bring any suggestions why human CNIHs were less efficient at supporting the functioning of Nha1 than Erv14, as all tested protein complexes were predicted with low confidence (ipTM values were typically ≈0.4–0.5, our unpublished data; not shown).

Human NHA2 Na^+^/H^+^ antiporter is a member of the CPA2 group of cation/proton antiporters (CPAs), in contrast to *S. cerevisiae* Nha1, which belongs to the CPA1 (Masrati et al., 2018). NHA2 is produced in multiple tissues, and its malfunctioning is connected to various pathologies, including hypertension or type 2 diabetes (Fuster et al., 2008; Liu et al., 2018; Xiang et al., 2007). This antiporter is necessary for insulin secretion in pancreatic β cells, blood‐pressure homeostasis, proper sperm motility and osteoclast differentiation in vitro (Battaglino et al., 2008; Chen et al., 2016; Deisl et al., 2013; Fuster et al., 2008; Ha et al., 2008; Kondapalli et al., 2017; Lee et al., 2008). However, so far, no information is available on NHA2's trafficking to its target membrane in cells. Our new yeast‐based screening system for cargoes of human cornichons revealed, for the first time, that a cornichon protein is required for an efficient plasma‐membrane targeting of the human NHA2 antiporter (Figures 5 and S8). The plasma‐membrane localization and functioning of NHA2, that is, its ability to export Na^+^ (Li^+^) cations in a pH‐dependent manner from cells, was supported by the presence of either yeast or human cornichons, with *Sc*Erv14 and its closest homologue CNIH4 being the most efficient (Figures 5 and S8). The lesser extent to which human cornichons improved the activity and localization of NHA2 when compared to *Sc*Erv14 might be the consequence of lower amounts of human cornichons present in yeast cells (Figure S5a) and/or their decreased ability to efficiently interact with yeast COPII‐coat proteins. AlphaFold3 models of NHA2 in complex with *Sc*Erv14 or human CNIHs were predicted with low ipTM values, similar to protein complexes with Nha1 discussed above (our unpublished data; not shown). Interestingly, cryogenic electron microscopy studies of bison or human NHA2 homodimers showed lipid molecules to be present at the interface between NHA2 monomers and also to influence the antiporter's activity (Jung et al., 2025; Matsuoka et al., 2022). In addition, experimental analyses of the complexes of human CNIH2/3 and AMPA receptors suggested the involvement of lipids in the formation of contacts between AMPARs and cornichons (Nakagawa, 2019; Yu et al., 2021; Zhang et al., 2021). Thus, we speculate that lipid molecules might be missing in our models of Na^+^/H^+^ antiporters in complex with cornichons, both at the interface between molecules of transporters and between the transporter and the cargo receptor.

Although it will be necessary to confirm the interaction of NHA2 with CNIHs directly in human cells, our results strongly suggest that NHA2 requires these proteins (particularly CNIH4) to be efficiently targeted to the membrane of its function. Data available from the Human Protein Atlas (www.proteinatlas.org) show that NHA2 as well as cornichons CNIH1 and CNIH4 are highly expressed in the stomach, duodenum, colon, and rectum— tissues that require tight pH regulation (Figure S10). Given that alkali‐metal‐cation/H^+^ antiporters of the CPA family, including NHA2, play important roles in pH homeostasis through their correct localization and activity, a high co‐expression of NHA2 with CNIH1 and CNIH4 in these tissues strongly supports a possible regulatory relationship.

## 4 CONCLUSIONS

The cornichon family of proteins, highly conserved in eukaryotic cells, is an important group of COPII‐coated vesicle cargo receptors, which helps numerous cargo proteins to exit the ER, proceed via the secretory pathway and reach their target membrane. The malfunctioning of four human cornichons, CNIH1‐4, has been linked to various pathologies, such as neurological disorders or cancer (Papouskova, Cerna, et al., 2024; Papouskova, Zimmermannova, & Sychrova, 2024). In this study, we aimed to evaluate the possibility of using *S. cerevisiae* cells as a tool to study the properties of human cornichons and extend our so‐far very limited knowledge of CNIHs' cargo repertoires. Being able to partially complement phenotypes related to the *S. cerevisiae* cornichon Erv14's functioning in the maintenance of cation homeostasis, human CNIHs proved to be functional in yeast cells. Moreover, human cornichons were able to support the functioning of *S. cerevisiae* Na^+^, K^+^/H^+^ antiporter Nha1, a cargo of the *Sc*Erv14 protein. Importantly, our experiments with cells producing human Na^+^/H^+^ antiporter NHA2 showed, for the first time, the protein's requirement of a cornichon cargo receptor for its efficient ER exit, thus proving NHA2 to be a newly identified binding partner of human cornichons. These results suggest that cornichon COPII‐coated vesicle cargo receptors are required for an efficient ER exit of Na^+^/H^+^ antiporters of both CPA1 and CPA2 subfamilies. A similar approach employing yeast cells can be used in the future for the discovery of other novel cargo proteins of human cornichons.

## 5 MATERIALS AND METHODS

### 5.1 Yeast strains, plasmids and growth media

The *S. cerevisiae* strains used in this study are listed in Table 1. To obtain BYT45 strains with human CNIH ORFs replacing *ScERV14* ORF in their genomes, we first amplified the corresponding cDNAs with codons optimized for their expression in yeasts (Figure S4) using the oligonucleotides listed in Table S2 and purchased pUC18‐based plasmids (Table S1) as templates. Further, human CNIH1, CNIH2, or CNIH4 cDNAs were inserted into the YEp352‐based plasmid pScSTL1‐kanMX (Table S1) by homologous recombination, replacing the *ScSTL1* gene. The obtained plasmids, pHsCNIH1‐kanMX, pHsCNIH2‐kanMX, or pHsCNIH4‐kanMX (Table S1) were used as templates for the amplification of integrative cassettes. The new yeast strains were then prepared by homologous recombination, and the proper insertion of human CNIH1, CNIH2, or CNIH4 ORFs behind the *ScERV14* promoter was confirmed by diagnostic PCRs.

The YEplac181‐based plasmids harboring either *ScERV14* or human CNIH1, CNIH2, or CNIH4 cDNAs tagged with the hemagglutinin sequence (Table S1) were prepared by homologous recombination in yeast cells. The cornichon ORFs were expressed under the control of the *ScERV14* promoter. A QuikChange XL Site‐Directed Mutagenesis kit (Agilent Technologies) and corresponding oligonucleotides (Table S2) were used to introduce point mutations into pScERV14‐HAt. The accuracy of the obtained plasmids was verified by sequencing.

Yeasts were routinely cultivated in YPD (1% yeast extract, 2% peptone, 2% glucose, Formedium), YNB (0.67% YNB without amino acids, Difco, 2% glucose), or YNB‐Pro (0.17% YNB without ammonium sulphate and amino acids, Difco, 0.1% proline, 2% glucose) media at 30°C. A filter‐sterilized low‐fluorescent YNB^pH^ medium (0.17% YNB without amino acids, ammonium sulphate, potassium, folic acid, and riboflavin, Formedium, 0.4% ammonium sulphate, 2% glucose, 50 mM KCl, pH adjusted to 5.8 with NH_4_OH) was used to determine intracellular pH levels in cells. YNB‐based media were supplemented with the oMM mixture of amino acids, which was developed to ensure optimum growth of BY4741 cells (Hanscho et al., 2012). The media were solidified by the addition of 2% or 3% agar.

### 5.2 Growth tests

Drop tests were used to monitor the growth of yeast cells on solid media in the presence of various concentrations of NaCl, KCl, LiCl, or tetramethylammonium (TMA^+^). Ten‐fold serial dilutions of fresh cell suspensions (OD_600_ = 2.0) were spotted on YPD, YNB, or YNB‐Pro (pH 4.0 adjusted with HCl) plates supplemented as indicated, and the growth was monitored for 2–7 days. A representative result of three independent experiments is presented.

To determine the growth of liquid cultures, cells were inoculated to OD_595_ = 0.01 into 100 μL of YNB‐Pro medium (pH 4.0 brought down with HCl) supplemented with LiCl as indicated in a 96‐well microplate and the increase in cell density was monitored in an ELx808 reader (BioTek) for 40 h as described previously (Maresova & Sychrova, 2007). Four technical replicates were used for each strain and set of growth conditions in individual experiments. The results are presented as means ± SEM of two to three independent measurements, as indicated in the text.

### 5.3 Membrane potential measurements

To estimate the relative plasma‐membrane potential of yeast cells, an assay based on the redistribution of the fluorescent dye 3,3′‐dipropylthiacarbocyanine iodide (diS‐C_3_(3); 0.1 mM stock solution in ethanol) was used (Gaskova et al., 1998; Kodedova & Sychrova, 2015). Cells grown in YPD to OD_600_ ≈ 0.8 were harvested, washed, and resuspended in 10 mM MES (pH 6.0 adjusted with triethanolamine) to OD_600_ = 0.2. 100 μL aliquots of cell suspensions were pipetted in duplicates to 96‐well clear flat‐bottomed polystyrene microplates with a nonbinding surface treatment (Corning). The fluorescence probe was added to a final concentration of 1 μM and fluorescence emission spectra (λ_ex_ = 531 nm, λ_em_ = 565–590 nm) were measured over 56 min at 2 min intervals in a Cytation 3 microplate reader (BioTek). The staining curves showing the time dependence of the maximum emission wavelength (λ_max_) were obtained as described previously (Kodedova & Sychrova, 2016). The presented values are means ± SEM of at least four independent measurements.

### 5.4 Intracellular pH measurements

For estimations of intracellular pH, BY4741^pHl^ and BY4741*erv14Δ* ^pHl^ cells (Table 1) producing pHluorin and harboring either an empty plasmid or plasmids ensuring the expression of *S* *c*Erv14 or *Hs*CNIH1/2/4 cDNAs (pScERV14‐HAt, pHsCNIH1‐HAt, pHsCNIH2‐HAt, pHsCNIH4‐HAt; Table S1), respectively, were cultivated in YNB^pH^ medium to OD_600_ ≈0.5. The fluorescence intensities (λ_ex_ = 395 and 475 nm, λ_em_ = 508 nm) were recorded using a Cytation 3 microplate reader (BioTek) according to (Albacar et al., 2021). A culture of BY4741 that did not produce pHluorin was grown in parallel, and the corresponding background fluorescence levels were subtracted from the fluorescence measured at each excitation wavelength. The ratios of emission intensity I_395nm_/I_475nm_ were used to obtain the values of intracellular pH as described previously (Zimmermannova et al., 2015). Eight technical replicates were measured for each strain within individual experiments. The presented values are means ± SEM of at least eight independent measurements.

### 5.5 Fluorescence microscopy

BYT45 and BYT45*erv14Δ* cells without or with CNIH1 or CNIH2 or CNIH4 cDNAs instead of *ScERV14* ORF that produced GFP‐tagged *Sc*Nha1 (at the C‐terminus) or human NHA2 (at the N‐terminus) were grown in YNB medium to OD_600_ ≈0.4. The fluorescence pattern of the GFP signal was viewed with an Olympus BX53 microscope equipped with a Cool LED light source with 460 nm excitation and 515 nm emission and a DP73 Olympus camera. Whole‐cell images were obtained with Nomarski optics.

To quantify the level of intracellular accumulation of GFP‐tagged alkali‐metal‐cation transporters, we counted the ratio of the corrected total cell fluorescence (CTCF) in the perinuclear ER (CTCF_pnER_) to the surface fluorescence in cells (CTCF_surface_) as described previously (McCloy et al., 2014; Papouskova, Cerna, et al., 2024; Papouskova, Zimmermannova, & Sychrova, 2024) using the software ImageJ (Schneider et al., 2012). With the polygon selections tool, we demarcated the perinuclear ER and surface area in individual cells separately, and the integrated density of the fluorescence signal was measured. Background fluorescence level was quantified in a region with no fluorescence next to the cells. The CTCF values were then obtained using the formula CTCF = Integrated density − (Selected cell area × Mean fluorescence of background) and CTCF_pnER_/CTCF_surface_ ratios were counted. Twenty cells were analyzed for each strain, and the results are presented as means ± SEM.

### 5.6 Intracellular sodium loss measurements

Sodium efflux was determined as described previously (Kinclova‐Zimmermannova et al., 2005; Velazquez et al., 2022). Cells grown in YNB (cells producing *Sc*Nha1‐GFP) or YNB‐Pro (cells with human NHA2) medium to OD_600_ ≈0.2–0.3 were harvested and incubated for 60 min in the same type of medium supplemented with 100 mM NaCl brought to pH 7.0 with NH_4_OH (YNB) or triethanolamine (YNB‐Pro) to preload them with sodium. Further, cells were collected, washed, and resuspended in an incubation buffer (10 mM Tris, 0.1 mM MgCl_2_, 2% glucose). The pH of the buffer was adjusted to 4.5 (cells with *Sc*Nha1‐GFP) or 4.0 (cells with human NHA2) with citric acid and Ca(OH)_2_. The buffer contained 10 mM KCl to prevent Na^+^ reuptake. Samples of cell suspensions were withdrawn over 40 min (cells with *Sc*Nha1‐GFP) or immediately after the transfer of cells to the incubation buffer and again after 60 min (cells producing human NHA2). Aliquots of cells were harvested by filtration, washed with 20 mM MgCl_2_, acid extracted, and the sodium content in samples was determined by atomic absorption spectrometry. The presented values are means ± SEM of at least three independent measurements.

### 5.7 Protein extraction and immunoblotting

Yeast cells transformed with plasmids encoding HA‐tagged cornichons were grown in liquid YNB medium to the exponential phase (OD_600_ ≈0.6–0.8), collected, and washed with deionized cold water. Obtained cell pellets were kept at −80°C. Total extracts of proteins were prepared as described previously (Horak & Wolf, 2001). The proteins were quantified using the RCDC protein assay (Bio‐Rad). 120 μg of protein extracts were separated by 10% SDS‐PAGE and transferred to nitrocellulose membranes (Trans‐Blot Turbo 0.2 μm nitrocellulose, Bio‐Rad) with a Trans‐Blot Turbo Transfer System (Bio‐Rad). After blocking, membranes were incubated overnight at 4°C with an anti‐HA rabbit polyclonal antibody (Sigma, dilution 1:1000). Washed membranes were subsequently incubated with a goat secondary anti‐rabbit IgG antibody tagged with a horseradish peroxidase (Sigma, dilution 1:5000). Immunoreactive proteins were then visualized with a Clarity Max Western ECL substrate kit (Bio‐Rad) in ChemiDoc visualizer (Bio‐Rad). Membrane staining with 0.2% Ponceau S was afterwards applied to check the protein loading. A representative result of two independent experiments is shown.

### 5.8 Sequence, structure and evolutionary conservation analysis

The phylogenetic tree and % identity of *Sc*Erv14 and human cornichons were obtained using the software Lasergene from DNASTAR. The alignment of *Sc*Erv14 and human CNIH1‐4 proteins was created with ClustalO and visualized using the ESPript 3.0 tool (Madeira et al., 2022; Robert & Gouet, 2014). The structural models of proteins were obtained from the AlphaFold protein structure database (Jumper et al., 2021; Varadi et al., 2022), and the images of proteins' structures and overlaps were acquired using the software PyMOL or directly from the AlphaFold database. AlphaFold Server (Abramson et al., 2024) with the default settings was applied to model the interaction of cornichons with Sec24 proteins. The UniProt database (The UniProt Consortium, 2025) numbers of the studied proteins are P53173 (*Sc*Erv14), O95406 (*Hs*CNIH1), Q6PI25 (*Hs*CNIH2), Q8TBE1 (*Hs*CNIH3), Q9P003 (*Hs*CNIH4), P40482 (*Sc*Sec24), and O95486 (*Hs*Sec24A). Human cornichon cDNA sequences were visualized with the software SnapGene.

To estimate the evolutionary conservation of *Sc*Erv14's amino‐acid residues, we generated a multiple‐sequence alignment of cornichon proteins. A homologue search was conducted using phmmer against the UniRef90 database (search parameters: E = 0.0001, incE = 0.0001, F1 = 0.0005, F2 = 0.00005, F3 = 0.0000005; Potter et al., 2018; Suzek et al., 2015) and *Sc*Erv14 as the query sequence. The results were filtered to include sequences with a range of 30% to 90% sequence identity compared to the query and coverage of 70%. Any fragmented or mutated sequences were excluded, and we manually removed a few sequences that introduced large gaps into the alignment. The multiple sequence alignment was generated using FAMSA (Deorowicz et al., 2016), encompassing 1879 sequences, including the four human cornichon sequences. The obtained alignment was then used in a ConSurf (Ashkenazy et al., 2016) analysis with the default settings.

Protein expression overviews of human CNIH1, CNIH4, and NHA2 in various organs/tissues were obtained from The Human Protein Atlas (proteinatlas.org, [89]).

### 5.9 Statistics

Data are presented either as a representative result or as means ± SEM as indicated in the text. Statistical analyses were performed in GraphPad Prism (version X) using one‐way ANOVA followed by Dunnett's multiple comparison test (* or ^$^ indicate significant differences to control cells with *ERV14* or without *erv14Δ*, respectively); *p* < 0.05 was considered significant (*/^$^ *p* < 0.05, **/^$$^ *p* < 0.01, ***/^$$$^ *p* < 0.001).

## AUTHOR CONTRIBUTIONS

**Karolína Kacovská:** Investigation. **Klára Papoušková:** Investigation; visualization; data curation; writing – original draft; writing – review and editing; validation; methodology. **Gal Masrati:** Investigation; data curation; writing – review and editing; software; visualization. **Paul Rosas‐Santiago:** Writing – review and editing; data curation; visualization. **Tereza Przeczková:** Investigation. **Veronika Žárská:** Investigation. **Nir Ben‐Tal:** Data curation; funding acquisition; supervision; writing – review and editing; software. **Olga Zimmermannová:** Conceptualization; investigation; funding acquisition; writing – review and editing; visualization; methodology; validation; formal analysis; project administration; data curation; supervision.

## FUNDING INFORMATION

The work of Olga Zimmermannova's group was supported by a GAČR grant 21‐08985S. The work of Nir Ben‐Tal's group was supported by grant 20250138 of the Israel Cancer Association, and the Abraham E. Kazan Chair in Structural Biology at Tel Aviv University. Gal Masrati was supported in part by a fellowship from the Edmond J. Safra Center for Bioinformatics at Tel‐Aviv University.

## Supporting information


**TABLE S1.** Plasmids used in this study.
**TABLE S2.** Oligonucleotides used in this study.
**FIGURE S1.** The influence of *Sc*Erv14's W68 mutations on the cornichon's ability to support localization and functioning of *S. cerevisiae* Na^+^, K^+^/H^+^ antiporter Nha1.
**FIGURE S3.** Models of interaction of human proteins CNIH1 or CNIH4 with Sec24A.
**FIGURE S4.** Sequences of cDNAs of human CNIH coding sequences used in this study.
**FIGURE S5.** Characterization of *S. cerevisiae* strains expressing human cornichons from plasmids.
**FIGURE S6.** Characterization of yeast strains with coding sequences of human cornichons integrated into the genome instead of the *ERV14* open reading frame.
**FIGURE S7.** The level of *Sc*Nha1‐GFP perinuclear ER accumulation in *S. cerevisiae* cells without or with *ERV14* or human CNIHs.
**FIGURE S8.** The influence of human cornichons on the functioning of human Na^+^/H^+^ antiporter NHA2 in *S. cerevisiae* cells.
**FIGURE S9.** The influence of human CNIH1 on the localization of *S. cerevisiae* K^+^ channel Tok1 in *S. cerevisiae* cells.
**FIGURE S10.** Protein expression overview of human CNIH1, CNIH4 and NHA2 in various human organs/tissues.

**FIGURE S2.** Multiple sequence alignment of 1879 sequences of cornichons from fungal, plant and animal species.

## Data Availability

The data that support the findings of this study are available from the corresponding author upon reasonable request.
