# Supplementary material for: Trafficking of the human Na+/H+ antiporter NHA2 to the plasma membrane requires cornichon COPII cargo receptors
Source: Protein Sci. 2026 Feb 12;35(3):e70492. doi: 10.1002/pro.70492 (PMC12895380; doi:10.1002/pro.70492)
Supplement: Supplementary file 1 — TABLE S1. Plasmids used in this study. TABLE S2. Oligonucleotides used in this study. FIGURE S1. The influence of ScErv14's W68 mutations on the cornichon's ability to support localization and functioning of S. cerevisiae Na+, K+/H+ antiporter Nha1. FIGURE S3. Models of interaction of human proteins CNIH1 or CNIH4 with Sec24A. FIGURE S4. Sequences of cDNAs of human CNIH coding sequences used in this study. FIGURE S5. Characterization of S. cerevisiae strains expressing human cornichons from plasmids. FIGURE S6. Characterization of yeast strains with coding sequences of human cornichons integrated into the genome instead of the ERV14 open reading frame. FIGURE S7. The level of ScNha1‐GFP perinuclear ER accumulation in S. cerevisiae cells without or with ERV14 or human CNIHs. FIGURE S8. The influence of human cornichons on the functioning of human Na+/H+ antiporter NHA2 in S. cerevisiae cells. FIGURE S9. The influence of human CNIH1 on the localization of S. cerevisiae K+ channel Tok1 in S. cerevisiae cells. FIGURE S10. Protein expression overview of human CNIH1, CNIH4 and NHA2 in various human organs/tissues. [file PRO-35-e70492-s002.pdf]

Trafficking of the human Na^+/H^+ antiporter NHA2 to the plasma membrane requires cornichon COPII cargo receptors

Karolína Kacovská[a], Klára Papoušková[a], Gal Masrati[b], Paul Rosas-Santiago[c], Tereza Przeczková[a], Veronika Žárská[a], Nir Ben-Tal[b], Olga Zimmermannová*[a]

SUPPLEMENTARY MATERIAL

[a] Laboratory of Membrane Transport, Institute of Physiology of the Czech Academy of Sciences, Prague 4, Czech Republic

[b] School of Neurobiology, Biochemistry and Biophysics, George S. Wise Faculty of Life Sciences, Tel-Aviv University, Tel-Aviv, Israel

[c] Instituto de Biotecnología, Universidad Nacional Autónoma de México, Av. Universidad 2001, Cuernavaca, Morelos, 62210, México

*Correspondence to Olga Zimmermannová: Laboratory of Membrane Transport, Institute of Physiology CAS, Videnska 1083, Prague 4 - Krc, 142 00, Czech Republic. Tel: +420 241 062 557. Fax: +420 241 062 488. E-mail: olga.zimmermannova@fgu.cas.cz

TABLE S1. Plasmids used in this study

Plasmid	Description	Source/reference
pUC18	Multi-copy empty vector	GenScript
pUC18-CNIH1	*Hs*CNIH1 in pUC18	GenScript
pUC18-CNIH2	*Hs*CNIH2 in pUC18	GenScript
pUC18-CNIH4	*Hs*CNIH4 in pUC18	GenScript
YEp352	Multi-copy empty vector (*URA3* marker)	[1]
pScSTL1-kanMX	*NHA1*^{P}*-ScSTL1-TPS1*^{T}*-loxP-kanMX-loxP* in YEp352	[2]
pHsCNIH1-kanMX	*NHA1*^{P}*-Hs*CNIH1-*TPS1*^{T}*-loxP- kanMX-loxP* in YEp352	This work
pHsCNIH2-kanMX	*NHA1*^{P}*-Hs*CNIH2-*TPS1*^{T}*-loxP- kanMX-loxP* in YEp352	This work
pHsCNIH4-kanMX	*NHA1*^{P}*-Hs*CNIH4-*TPS1*^{T}*-loxP- kanMX-loxP* in YEp352	This work
pHsNHA2t	*NHA1*^{P}*-Hs*NHA2-*TPS1*^{T} in YEp352	[3]
pGFP-HsNHA2t	*NHA1*^{P}*-GFP-Hs*NHA2-*TPS1*^{T} in YEp352	[3]
pGRU1	Multi-copy empty vector with GFP (*URA3* marker)	B. Daignan-Fornier, NCBI Acc.No. AJ249649
pNHA1-985GFP	*NHA1*^{P}*-ScNHA1* in pGRU1	[4]
YEplac181	Multi-copy empty vector (*LEU2* marker)	[5]
pScERV14-HAt	*ERV14*^{P}*-ScERV14-HA-TPS1*^{T} in YEplac181	This work
pScERV14-W68A-HAt	pScERV14-HA, *ScERV14* mutated to encode Erv14-W68A	This work
pScERV14-W68P-HAt	pScERV14-HA, *ScERV14* mutated to encode Erv14-W68P	This work
pHsCNIH1-HAt	*ERV14*^{P}*-Hs*CNIH1-*HA-TPS1*^{T} in YEplac181	This work
pHsCNIH2-HAt	*ERV14*^{P}*-Hs*CNIH2-*HA-TPS1*^{T} in YEplac181	This work
pHsCNIH4-HAt	*ERV14*^{P}*-Hs*CNIH4-*HA-TPS1*^{T} in YEplac181	This work

^{P}, promoter; ^{T}, terminator; *HA*, hemagglutinin sequence.

Table S1 references

1. Hill JE, Myers AM, Koerner TJ, Tzagoloff A. Yeast/*E. coli* shuttle vectors with multiple unique restriction sites. Yeast. 1986;2:163-167.
2. Duskova M, Ferreira C, Lucas C, Sychrova H. Two glycerol uptake systems contribute to the high osmotolerance of *Zygosaccharomyces rouxii*. Mol Microbiol. 2015;97:541-559.
3. Velazquez D, Prusa V, Masrati G, et al. Allosteric links between the hydrophilic N-terminus and transmembrane core of human Na^+/H^+ antiporter NHA2. Protein Sci. 2022;31:e4460.
4. Kinclova O, Ramos J, Potier S, Sychrova H. Functional study of the *Saccharomyces cerevisiae* Nha1p C-terminus. Mol Microbiol. 2001;40:656-668.
5. Gietz RD, Sugino A. New yeast-*Escherichia coli* shuttle vectors constructed with in vitro mutagenized yeast genes lacking six-base pair restriction sites. Gene. 1988;74:527-534.

TABLE S2. Oligonucleotides used in this study

**Oligonucleotide**	**Sequence (5´- 3´)**
**Oligonucleotides used for the preparation of pHsCNIH1/2/4-kanMX plasmids**	
CNIH1-FS1*	TGTACATTATAAAAAAAAATCCTGAACTTAGCTAGATATTATGGCTTTTACTTTCGCTGC
CNIH1-RS1	GGACCAGGAATAGACGATCGTCTCATTTGCATCGGGTTCACTATGAAGAAACTAAAACATAG
CNIH2-RS2	CGGACCAGGAATAGACGATCGTCTCATTTGCATCGGGTTCACTAAGAAACTAAAGTGTAAAC
CNIH4-FS2	TGTACATTATAAAAAAAAATCCTGAACTTAGCTAGATATTATGGAAGCTGTTGTTTTCGTTTTC
CNIH4-RS2	GGACCAGGAATAGACGATCGTCTCATTTGCATCGGGTTCACTAATCATTAATCAATGCTAAAATC
**Oligonucleotides used for the amplification of CNIH1/2/4-kanMX integrative cassettes**	
IntgCNIH1-F1*	CTGCAATTAAAGTAAAGTAAAAAAATTAAGAATAAAAAGAAAAATGGCTTTTACTTTCGCTGC
IntgCNIH4-F1	CTGCAATTAAAGTAAAGTAAAAAAATTAAGAATAAAAAGAAAAATGGAAGCTGTTGTTTTCGTTTTC
IntgkanMX-R1	CTTGGCCCTTCAGTCTTCTTTGGATTTCAATGTCTTGTTGGAGCATAGGCCACTAGTGGATCTG
**Oligonucleotides used for the preparation of pScERV14-HA and pHsCNIH1/2/4-HA plasmids**	
pERV14_F	TGACCATGATTACGCCAAGCTTGCATGCCTGCAGGTCGACAACCAGTTGTCAAAACAGTTTATCAG
ERV14_HA_R	CTCATTTGCATCGGGTTCACTAAGCGTAATCTGGAACATCGTATGGGTAGAAGTCATCACCACTTTCAGC
pERV14_R1	CAATGCCAACATGTAACAGAATGCAGCGAAAGTAAAAGCCATTTTTCTTTTTATTCTTAATTTTTTTAC
pERV14_R2	CAATGTCAACATGTAACAGAATGCAGCGAAAGTAAAAGCCATTTTTCTTTTTATTCTTAATTTTTTTAC
pERV14_R4	CAACAATCCAACAAAGAGAAAACGAAAACAACAGCTTCCATTTTTCTTTTTATTCTTAATTTTTTTAC
CNIH1_F1*	GCAATTAAAGTAAAGTAAAAAAATTAAGAATAAAAAGAAAAATGGCTTTTACTTTCGCTGC
CNIH1_HA_R	CATTTGCATCGGGTTCACTAAGCGTAATCTGGAACATCGTATGGGTATGAAGAAACTAAAACATAGATC
CNIH2_HA_R	CATTTGCATCGGGTTCACTAAGCGTAATCTGGAACATCGTATGGGTAAGAAACTAAAGTGTAAACCATTG
CNIH4_F1	CAATTAAAGTAAAGTAAAAAAATTAAGAATAAAAAGAAAAATGGAAGCTGTTGTTTTCGTTTTC
CNIH4_HA_R	GCATCGGGTTCACTAAGCGTAATCTGGAACATCGTATGGGTAATCATTAATCAATGCTAAAATCATAG
TPS1_R	GTAAAACGACGGCCAGTGAATTCGAGCTCGGTACCCGGGGATCCTGTTTCGAAGAAGAGATCAGC
EHA-TPS1-F	GTGATGACTTCTACCCATACGATGTTCCAGATTACGCTTAGTGAACCCGATGCAAATGAGACGATCG
C1HA_TPS1_F	GTTTTAGTTTCTTCATACCCATACGATGTTCCAGATTACGCTTAGTGAACCCGATGCAAATGAGACGATCG
C2HA_TPS1_F	CACTTTAGTTTCTTACCCATACGATGTTCCAGATTACGCTTAGTGAACCCGATGCAAATGAGACGATCG
C4HA_TPS1_F	GATTTTAGCATTGATTAATGATTACCCATACGATGTTCCAGATTACGCTTAGTGAACCCGATGCAAATGAGAC
**Oligonucleotides used for site-directed mutagenesis of *ScERV14***	
ScERV14_W68A_F	CTGAACGGTTACGCGTTTGTATTTTTA
ScERV14_W68A_R	TAAAAATACAAACGCGTAACCGTTCAG
ScERV14_W68P_F	CTTACTGAACGGTTACCCGTTTGTATTTTTATTG
ScERV14_W68P_R	CAATAAAAATACAAACGGGTAACCGTTCAGTAAG
**Diagnostic oligonucleotides**	
PromErv14_F1	CTATCACATCACGCTTTCAC
ScErv14_R1	GCTAGAACTGGTAAGTTC
ScErv14_F1	GATGCTACCGAAATATTC
ScErv14 D-R1	TTTCACAGTCATGCTCACCC
CNIH1_R1	CCTTTTGACAGTATGCCAAG
CNIH2_R1	CAACTTTCTCAACAAACAACAG
CNIH4_R1	CACCCATATTACCAGATGGAA
kanMX_F1	CATTTGATGCTCGATGA

* The same oligonucleotide was used for cloning of human CNIH1 or CNIH2.

FIGURE S1

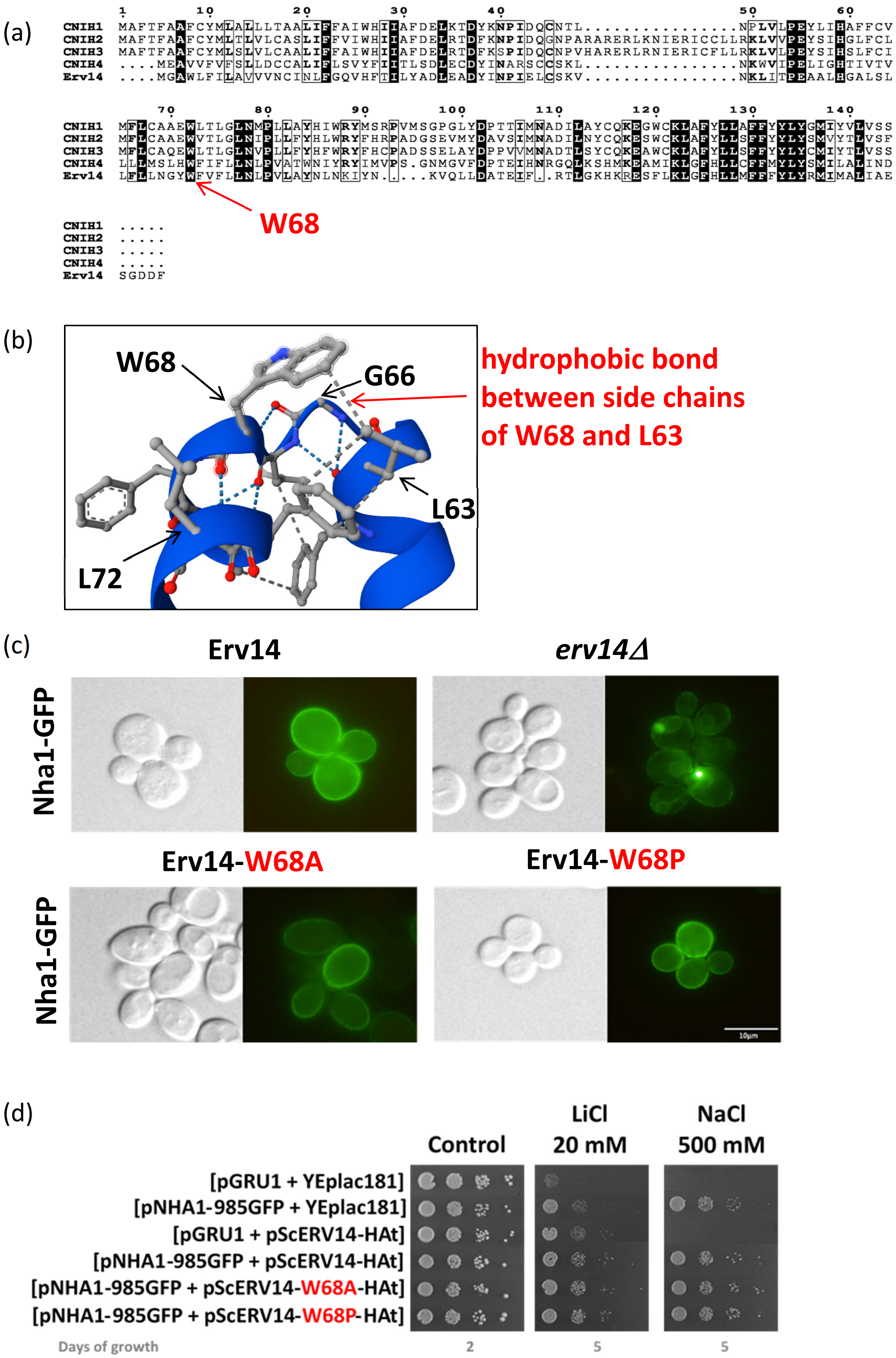
(a)
1 10 20 30 40 50 60
CNIH1 MAFTFAAFCYMLALLLTAALIFFAIWHIIAFDELKTDYKNPIDQCNTL................NPLVLPEYLIHAFFCV
CNIH2 MAFTFAAFCYMLTLVLCASLIFFVIWHIIAFDELRTDFKNPIDQGNPARARERLKNIERICCLLRKLVVPEYSIHGLFCL
CNIH3 MAFTFAAFCYMLSLVLCAALIFFAIWHIIAFDELRTDFKSPIDQCNPVHARERLRNIERICFLLRKLVLPEYSIHSLFCI
CNIH4 ....MEAVVFVFSLLDCCALIFLSVYFIITLSDLECDYINARSCCSKL................NKWVIPELIGHTIVTV
Erv14 ....MGAWLFILAVVVNCINLFGQVHFTILYADLEADYINPIELCSKV................NKLITPEAALHGALSL
70 80 90 100 110 120 130 140
CNIH1 MFLCAAEWLTLGLNMPLLAYHIWRYMSRPVMSGPGLYDPTTIMNADILAYCQKEGWCKLAFYLLAFFYYLYGMIYVLVSS
CNIH2 MFLCAAEWVTLGLNIPLLFYHLWRYFHRPADGSEVMYDAVSIMNADILNYCQKESWCKLAFYLLSFFYYLYSMVYTLVSF
CNIH3 MFLCAQEWLTLGLNVPLLFYHFWRYFHCPADSSELAYDPPVVMNADTLSYCQKEAWCKLAFYLLSFFYYLYCMIYTLVSS
CNIH4 LLLMSLHWFIFLLNLPVATWNIYRYIMVPS.GNMGVFDPTEIHNRGQLKSHMKEAMIKLGFHLLCFFMYLYSMILALIND
Erv14 LFLLNGYWFVFLLNLPVLAYNLNKIYN.....KVQLLDATEIF..RTLGKHKRESFLKLGFHLLMFFFYLYRMIMALIAE
W68
CNIH1 .....
CNIH2 .....
CNIH3 .....
CNIH4 .....
Erv14 SGDDF
(b)
W68
G66
L63
L72
hydrophobic bond between side chains of W68 and L63
(c)
Erv14
erv14Δ
Nha1-GFP
Erv14-W68A
Erv14-W68P
Nha1-GFP
(d)
Control
LiCl 20 mM
NaCl 500 mM
[pGRU1 + YEplac181]
[pNHA1-985GFP + YEplac181]
[pGRU1 + pScERV14-HAt]
[pNHA1-985GFP + pScERV14-HAt]
[pNHA1-985GFP + pScERV14-W68A-HAt]
[pNHA1-985GFP + pScERV14-W68P-HAt]
Days of growth
2
5
5

FIGURE S1 The influence of *Sc*Erv14´s W68 mutations on the cornichon's ability to support localization and functioning of *S. cerevisiae* Na^+, K^+/H^+ antiporter Nha1 tagged with GFP at the C-terminus in yeast cells. (a) Multiple sequence alignment of the studied cornichons. *Sc*Erv14´s amino-acid residue W68 is highlighted. (b) A detail of Erv14´s structural model, showing W68 and its closest surroundings. Amino-acid residues that are predicted to non-covalently interact with W68 are highlighted. Mutations W68A or W68P in *Sc*Erv14 changed neither the localization (c) nor the functioning (d) of Nha1-GFP. BYT45*erv14Δ* cells (*nha1Δ ena1-5Δ erv14Δ*) were transformed with the multi-copy plasmid encoding Nha1-GFP and the plasmid encoding none or indicated versions of *Sc*Erv14 (tagged with HA at the C-terminus). In (c), transformants were observed under a fluorescence microscope (right), and a Nomarski prism was used for whole-cell imaging (left). While the Nha1-GFP is partially stacked in the perinuclear ER in cells lacking Erv14 (*erv14Δ*), it is properly targeted to the plasma membrane in cells with any version of Erv14. In (d), growth of Nha1-GFP producing BYT45*erv14Δ* cells on plates supplemented with salts as indicated. Cells with empty vectors (pGRU1, YEplac181) were used as controls. Cells expressing Nha1-GFP together with any version of *Sc*Erv14 grew the same and better in the presence of high LiCl or NaCl than cells without *Sc*Erv14. Results of a representative experiment of at least three independent repetitions are shown.

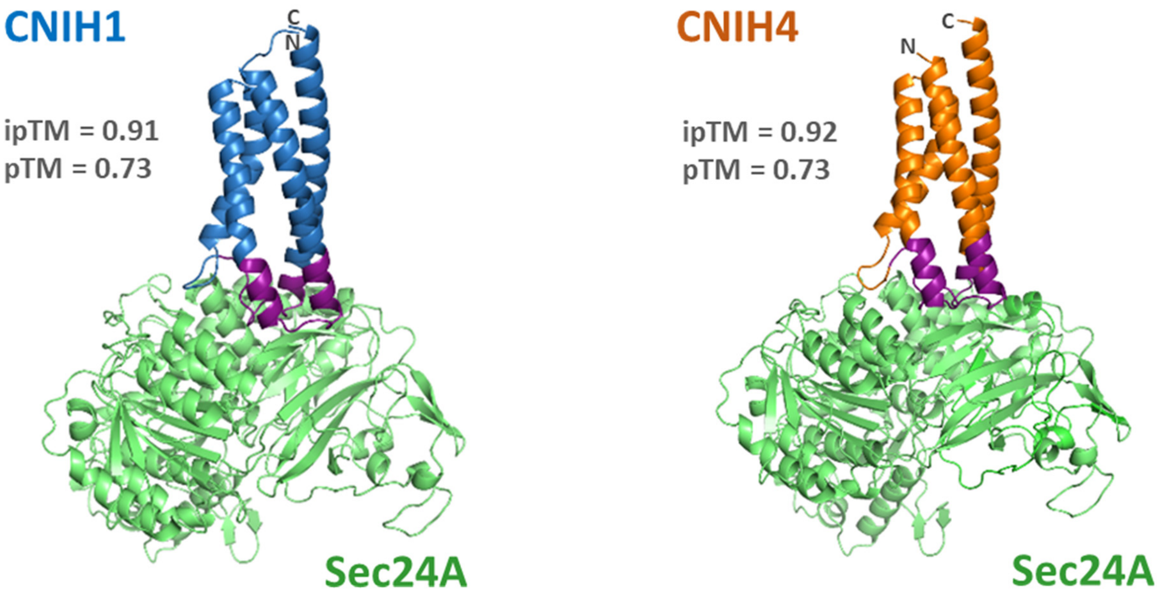

FIGURE S3 Models of interaction of human proteins CNIH1 or CNIH4 with Sec24A. The models were created using AlphaFold 3 and demonstrated a high level of confidence, with pTM and ipTM measures above 0.7 and 0.9, respectively, on the 0-through-1 scale. Sec24A is coloured in green, cornichons in blue (CNIH1) or orange (CNIH4), with evolutionarily conserved regions highlighted in violet. The intrinsically disordered N-terminal portion of Sec24A (aa 1-345) is not shown.

CNIH1 (aa 1-144)

ATGGCTTTTACTTTCGCTGCATTCTGTTACATGTTGGCATTGTTGTTGACAGCTGCATTGATTTTCTTTGCTATCTGGCATATCATCGCATTCGATGAATTGAAGACTGATTACAAGAAT

M A F T F A A F C Y M L A L L L T A A L I F F A I W H I I A F D E L K T D Y K N

1 5 10 15 20 25 30 35 40

CCAATCGATCAATGTAACACATTGAACCCATTGGTTTTGCCAGAATACTTAATCCATGCTTTCTTTTGTGTTATGTTTTTATGTGCTGCAGAATGGTTGACTTTGGGTTTGAACATGCCA

P I D Q C N T L N P L V L P E Y L I H A F F C V M F L C A A E W L T L G L N M P

45 50 55 60 65 70 75 80

TTGTTGGCTTACCATATCTGGAGATACATGTCTAGACCAGTTATGTCAGGTCCAGGTTTGTACGATCCAACTACAATCATGAACGCTGATATCTTGGCATACTGTCAAAAGGAAGGTTGG

L L A Y H I W R Y M S R P V M S G P G L Y D P T T I M N A D I L A Y C Q K E G W

85 90 95 100 105 110 115 120

TGTAAGTTGGCTTTCTACTTATTGGCTTTCTTTTATTACTTGTACGGTATGATCTATGTTTTAGTTTCTTCATAG

C K L A F Y L L A F F Y Y L Y G M I Y V L V S S *

125 130 135 140 145

CNIH2 (aa 1-159)

ATGGCTTTTACTTTCGCTGCATTCTGTTACATGTTGACATTGGTTTTGTGTGCTTCATTGATTTTCTTTGTTATTTGGCATATTATTGCATTCGATGAATTGAGAACTGATTTCAAGAAT

M A F T F A A F C Y M L T L V L C A S L I F F V I W H I I A F D E L R T D F K N

1 5 10 15 20 25 30 35 40

CCAATTGATCAAGGTAATCCAGCTAGAGCAAGAGAAAGATTGAAAAATATCGAAAGAATCTGTTGTTTGTTGAGAAAGTTGGTTGTTCCAGAATACTCTATCCATGGTTTATTTTGTTTG

P I D Q G N P A R A R E R L K N I E R I C C L L R K L V V P E Y S I H G L F C L

45 50 55 60 65 70 75 80

ATGTTTTTATGTGCTGCAGAATGGGTTACATTAGGTTTGAACATCCCATTGTTGTTTTATCATTTGTGGAGATACTTCCATAGACCAGCTGATGGTTCAGAAGTTATGTACGATGCTGTT

M F L C A A E W V T L G L N I P L L F Y H L W R Y F H R P A D G S E V M Y D A V

85 90 95 100 105 110 115 120

TCTATCATGAACGCAGATATCTTGAACTACTGTCAAAAGGAATCATGGTGTAAGTTGGCATTCTACTTGTTGTCTTTCTTTTATTACTTGTATTCAATGGTTTACACTTTAGTTTCTTAG

S I M N A D I L N Y C Q K E S W C K L A F Y L L S F F Y Y L Y S M V Y T L V S *

125 130 135 140 145 150 155 160

CNIH4 (aa 1-139)

ATGGAAGCTGTTGTTTTCGTTTTCTCTTTGTTGGATTGTTGTGCATTGATTTTCTTGTCTGTTTACTTCATCATTACTTTATCAGATTTGGAATGTGATTACATCAACGCTAGATCTTGT

M E A V V F V F S L L D C C A L I F L S V Y F I I T L S D L E C D Y I N A R S C

1 5 10 15 20 25 30 35 40

TGTTCAAAGTTGAATAAGTGGGTTATTCCAGAATTGATCGGTCATACTATCGTTACAGTTTTGTTGTTGATGTCATTGCATTGGTTCATTTTCTTGTTGAATTTGCCAGTTGCAACTTGG

C S K L N K W V I P E L I G H T I V T V L L L M S L H W F I F L L N L P V A T W

45 50 55 60 65 70 75 80

AACATCTATAGATACATCATGGTTCCATCTGGTAATATGGGTGTTTTCGATCCAACAGAAATCCATAACAGAGGTCAATTGAAGTCACATATGAAGGAAGCTATGATCAAGTTGGGTTTC

N I Y R Y I M V P S G N M G V F D P T E I H N R G Q L K S H M K E A M I K L G F

85 90 95 100 105 110 115 120

CATTTGTTGTGTTTCTTTATGTATTTGTACTCTATGATTTTAGCATTGATTAATGATTAG

H L L C F F M Y L Y S M I L A L I N D *

125 130 135 140

FIGURE S4 Sequences of cDNAs of human CNIH coding sequences used in this work. Codons were optimised for the expression in *S. cerevisiae*.

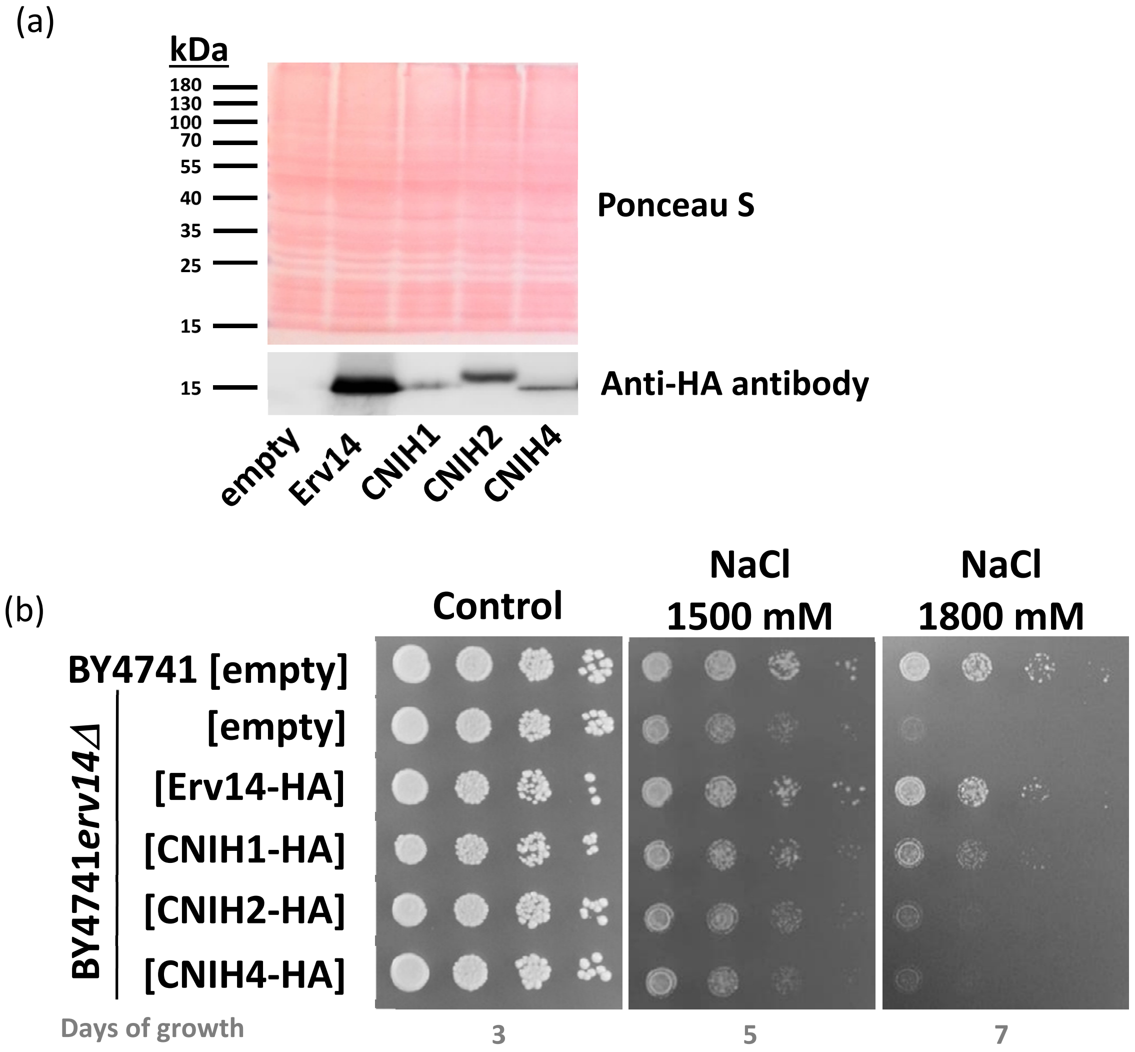

FIGURE S5 Characterization of *S. cerevisiae* strains expressing human cornichons from plasmids. (a) Immunodetection of HA-tagged cornichon proteins produced in BYT45*erv14Δ* cells (*nha1Δ ena1-5Δ erv14Δ*) transformed with multi-copy plasmids encoding indicated yeast (Erv14) or human (CNIH1, CNIH2, CNIH4) cornichons. The cornichons were detected with an anti-HA antibody, and the proper protein loading was checked by staining the membrane with Ponceau S. A representative experiment of two independent repetitions is shown. (b) Growth of BY4741 cells (with *ERV14* in the genome) or without the gene (BY4741*erv14Δ*) transformed either with the empty vector or with multi-copy plasmids encoding indicated yeast (Erv14) or human (CNIH1, CNIH2, CNIH4) cornichons (tagged with HA) on plates supplemented with indicated amount of NaCl. Results of a representative experiment of three independent repetitions are shown.

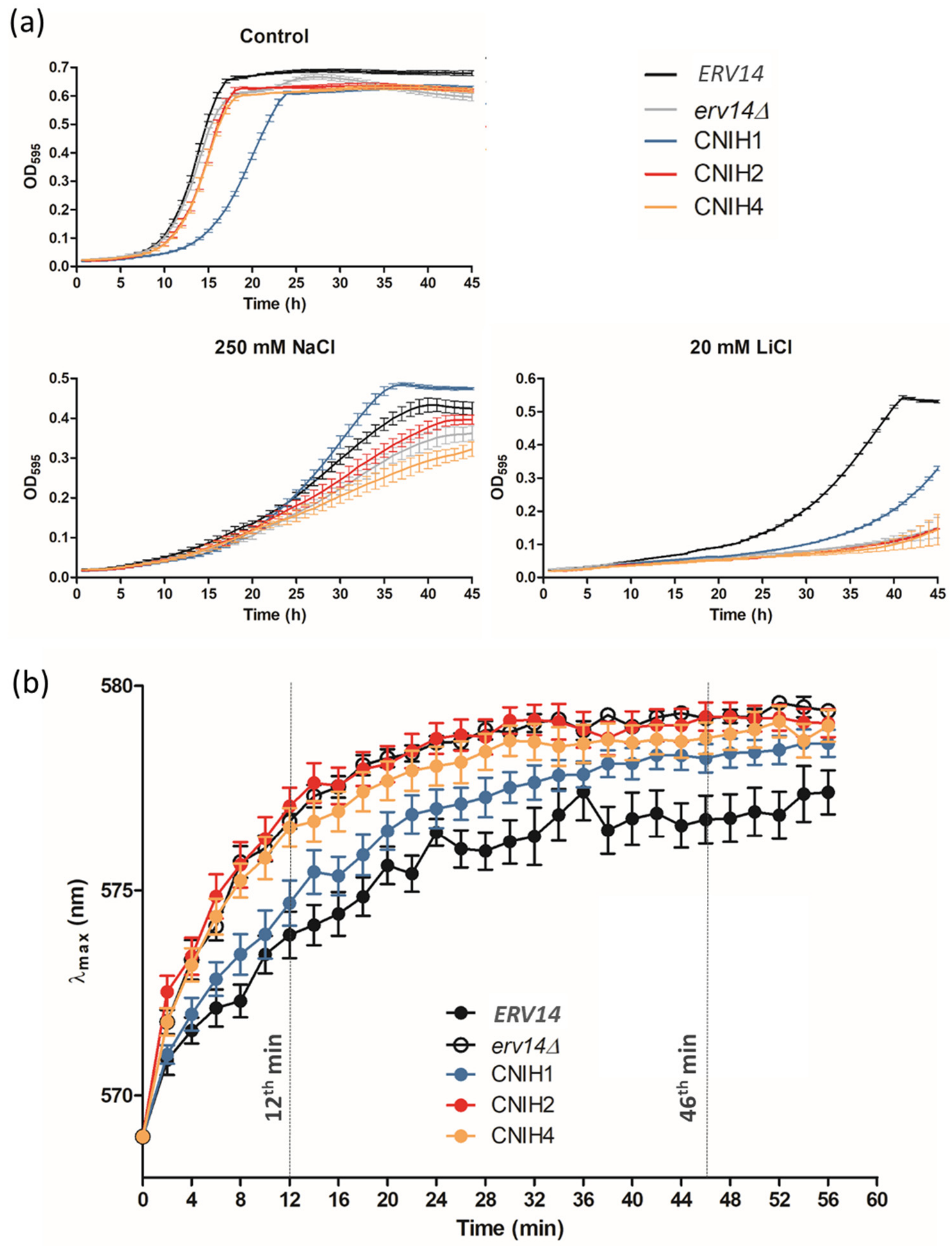

FIGURE S6 Characterization of yeast strains with coding sequences of human cornichons integrated into the genome instead of the *ERV14* open reading frame. (a) Growth of BYT45 (*nha1Δ ena1-5Δ*) cells with *ERV14* or its derivatives without the *ERV14* gene (*erv14Δ*) or with *ERV14* replaced with human cDNAs encoding CNIH1, CNIH2 or CNIH4 in the genome in liquid media without or supplemented with salts as indicated. Data represent the mean values ± SEM from three (control, NaCl) or two (LiCl) independent experiments (with four technical replicates each). (b) Relative membrane potential of the same strains as in (a). Entire diS-C_3(3) fluorescent probe staining curves are shown, and positions of values shown as columns in Fig. 3b, c, measured at 12 and 46 min, respectively, after the addition of the probe, are highlighted. Data represent the mean values ± SEM from four independent experiments (with two technical replicates each).

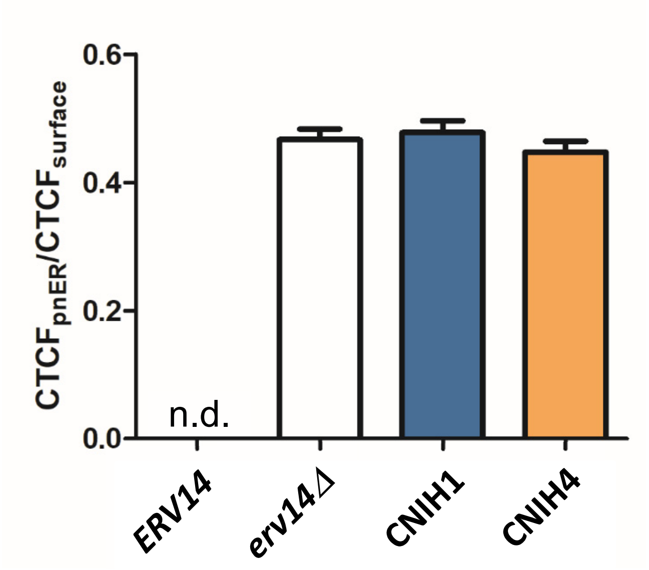

FIGURE S7 The level of *Sc*Nha1-GFP perinuclear ER (pnER) accumulation in BYT45 (*nha1Δ ena1-5Δ*) cells with *ERV14* or without the gene (*erv14Δ*) or strains with *ERV14* replaced by human CNIH1 or CNIH4 cDNAs in the genome expressed as the ratios of $CTCF_{pnER}/CTCF_{surface}$.The fluorescence signal in twenty cells was analysed for each strain. Data represent mean values ± SEM. n.d., value not determined.

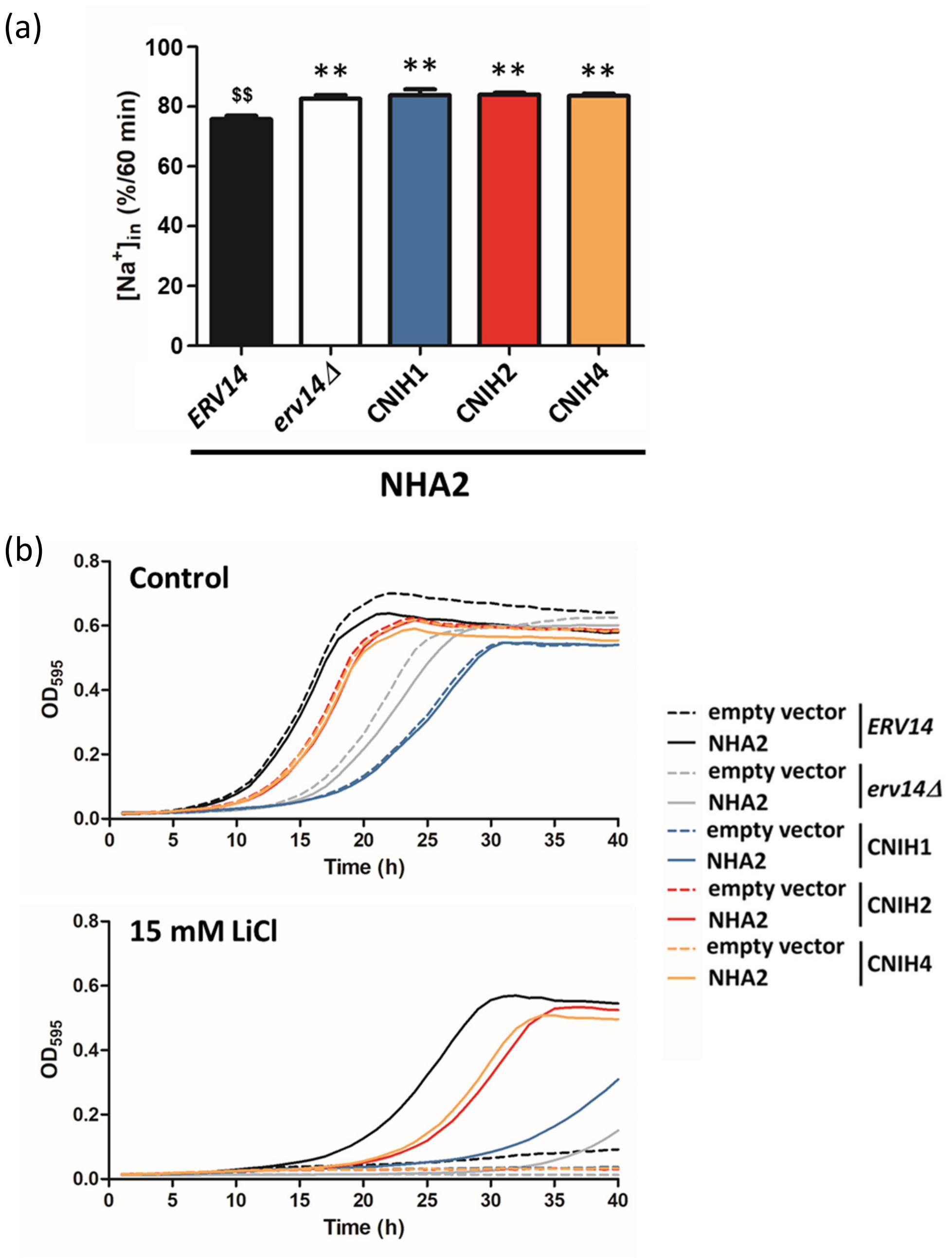

FIGURE S8 The influence of human cornichons on the functioning of human Na^+/H^+ antiporter NHA2 in *S. cerevisiae* cells. (a) Na^+ content in NHA2-producing BYT45 derivatives with *ERV14* or without the gene (*erv14Δ*) or with *ERV14* replaced with human CNIH cDNAs in the genome. Columns represent the amount of Na^+ in cells after 60 min of measurements of Na^+ efflux. Results are expressed as % of the value measured at time 0. The average initial amount of Na^+ in cells was 186.09 ± 6.15 nmol/mg dry weight. Symbols * or $^{\$}$ indicate statistically significant differences to control cells with *ERV14* or without *erv14Δ*, respectively, $**/^{\$\$}$ $p < 0.01$. (b) Growth of human NHA2-producing BYT45 cells as in (a) in liquid media supplemented with LiCl as indicated. The same strains transformed with the empty vector were used as controls. Means of three independent experiments ± SEM are shown.

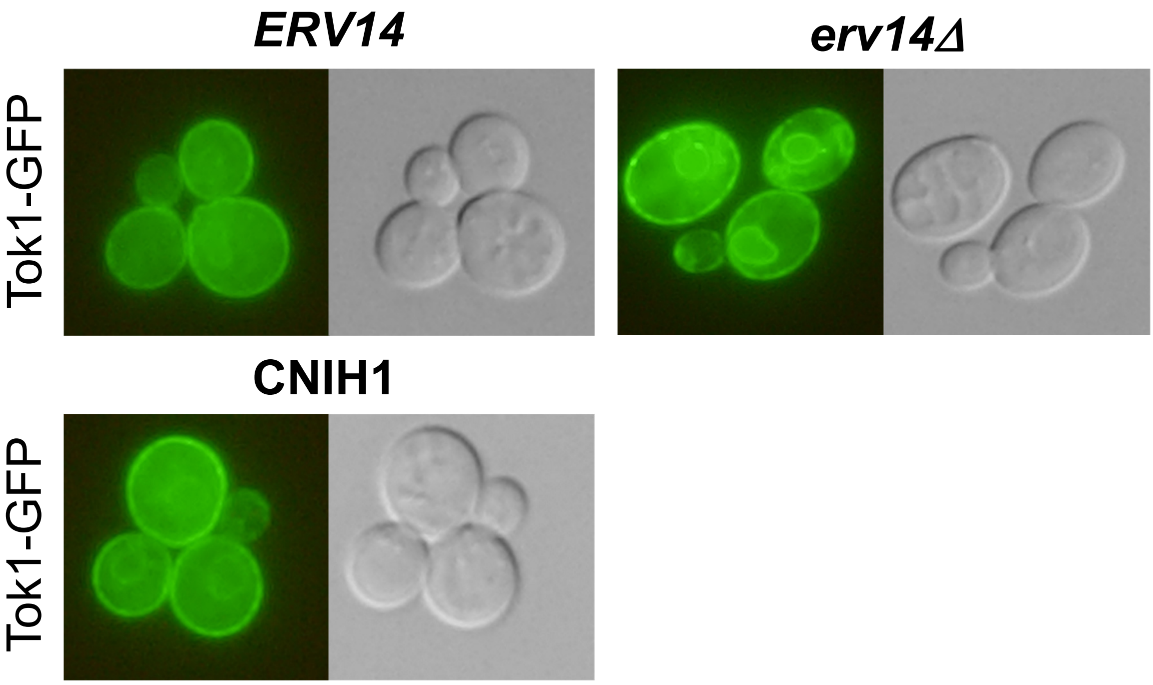

FIGURE S9 Human CNIH1 improves the localization of *S. cerevisiae* K^+ channel Tok1 in yeast cells. BYT123*erv14* yeast cells lacking the gene *ERV14* and three K^+ specific transporters (*trk1Δ trk2Δ tok1Δ erv14Δ*) [1] contained either the empty vector (indicated as *erv14Δ*) or expressed *Sc*Erv14 (*ERV14*) or human CNIH1 (CNIH1) form plasmids pScERV14-HA or pHsCNIH1-HA (Table S1). K^+ channel Tok1 tagged with GFP at the C-terminus was expressed in cells from a multi-copy plasmid (pY-TOK1-GFP) [1]. Transformants were observed under a fluorescence microscope (left), and a Nomarski prism was used for whole-cell imaging (right). While the Tok1-GFP is partially stacked in the perinuclear ER in cells lacking Erv14 (*erv14Δ*), it is more efficiently targeted to the plasma membrane in cells with *Sc*Erv14 and human CNIH.

Fig. S9 reference:

1. Zimmermannova O, Felcmanova K, Rosas-Santiago, P, et al. Erv14 cargo receptor participates in regulation of plasma-membrane potential, intracellular pH and potassium homeostasis via its interaction with K^+-specific transporters Trk1 and Tok1. Biochim Biophys Acta Mol Cell Res. 2019;1866:1376-1388. https://doi.org/10.1016/j.bbamcr.2019.05.005.

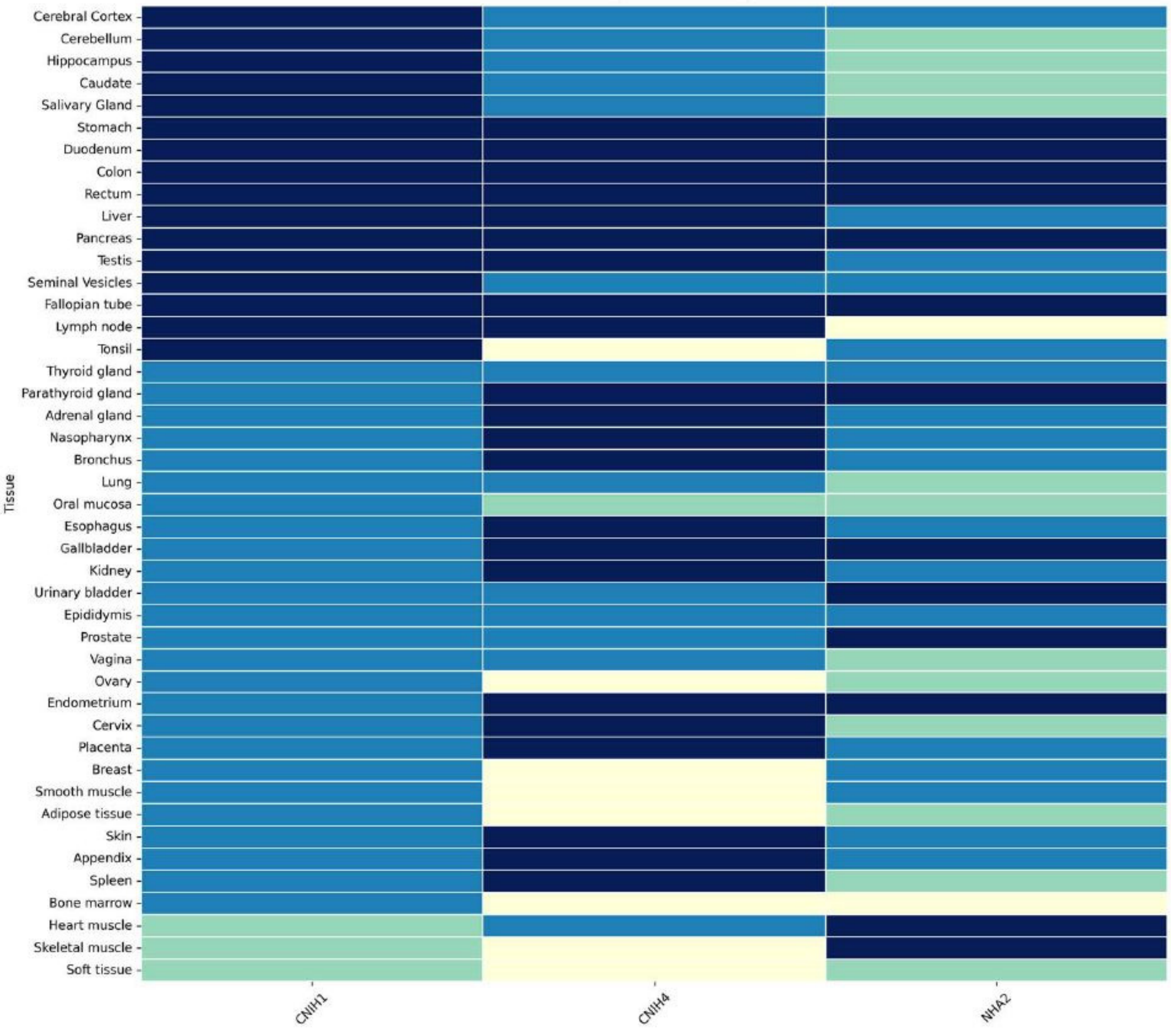

FIGURE S10 Protein expression overview of human CNIH1, CNIH4 and NHA2 in various human organs/tissues. Dark blue, high expression; light blue, medium expression; green, low expression; beige, protein not detected.
